# Combined Chemical–Physical Modification of Coal Gangue Concrete: ITZ Densification and Enhanced Wetting–Drying Resistance

**DOI:** 10.3390/ma19204332

**Published:** 2026-10-15

**Authors:** Feng Wei, Yuedan Li, Shilong Zhang, Yangguang Yan, Yugen Li, Fei Zhang, Gaoyang Li, Liangliang Bao

**Affiliations:** 1 Architectural Engineering Institute, Yulin University, Yulin 719000, China; 2 College of Water Resources and Hydropower, Xi’an University of Technology, Xi’an 710048, China

**Keywords:** coal gangue concrete, combined chemical–physical modification, wetting–drying cycles, durability

## Abstract

To investigate a combined chemical–physical modification strategy for improving the wetting–drying resistance of coal gangue concrete (CGC), CGC was prepared by replacing 25 wt.% of natural coarse aggregate with coal gangue coarse aggregate. Sodium silicate (8 wt.%) was used for chemical activation, while waterborne epoxy resin (3, 6, and 9 wt.%) was introduced to achieve physical sealing. The modification mechanism was investigated through mechanical tests, relative dynamic modulus measurements, X-ray diffraction (XRD), and scanning electron microscopy (SEM). The results showed that the combined effects of chemical activation and physical sealing contributed to improved resistance to deterioration under wetting–drying cycles, while the modification performance was also dependent on the resin dosage. Among the tested mixtures, CSM–6–8, representing the combined-modification group treated with a 6 wt.% aqueous epoxy modifying solution and an 8 wt.% sodium silicate solution, exhibited the best overall performance, with compressive and flexural strengths of 50.5 MPa and 10.9 MPa, respectively, after 60 wetting–drying cycles. After 120 cycles, its compressive and flexural strengths remained 11.92% and 33.70% higher than those of SM–0–8, respectively, indicating a sustained improvement in wetting–drying resistance. Its relative dynamic modulus reached a peak value of 1.05 after 28 cycles and decreased by only 3.4% relative to this peak after 90 cycles. Microstructural analyses suggested that sodium silicate modification may promote the formation of additional gel-like hydration products, while the cured epoxy resin may contribute to the development of a relatively continuous surface coating, with these effects jointly contributing to the densification of the pore structure and interfacial transition zone (ITZ), thereby reducing moisture transport and delaying deterioration. The better performance of the 6 wt.% resin dosage among the tested levels was associated with a favorable balance between coating development and the tendency for resin agglomeration at higher dosages. This study provides support for the design of coal gangue concrete with improved wetting–drying resistance and promotes its low-carbon utilization.

## 1. Introduction

Rapid industrialization has contributed substantially to economic development; however, it has also triggered a range of environmental challenges, such as the depletion of natural resources, the large-scale accumulation of industrial by-products, and soil erosion [1,2]. Coal gangue, a by-product of coal mining, has become one of the largest accumulated solid wastes in China [3]. Meanwhile, the growing demand for concrete in the construction industry has further intensified the depletion of natural aggregate resources [4]. To alleviate these environmental pressures and promote sustainable development, the resource utilization of industrial by-products has been increasingly recognized as an effective and sustainable strategy [5,6,7]. Incorporating coal gangue as aggregate in concrete can reduce the reliance of concrete production on natural aggregates while improving the resource utilization efficiency of coal gangue [8]. However, compared with natural aggregates, coal gangue aggregates (CGAs) generally exhibit several inherent limitations, such as relatively low strength, high water absorption, well-developed pore structures, loose surfaces, and considerable variability in properties [9]. The direct incorporation of CGA into concrete may weaken the mechanical properties and durability of concrete due to these inherent characteristics, thereby limiting their engineering applications. Accordingly, aggregate modification has become an important strategy for improving the surface characteristics and pore structure of CGA, as well as their interfacial bonding with cement paste [10]. However, owing to the intrinsic defects of CGA, the performance of CGC remains generally inferior to that of conventional concrete [11,12,13].

Northwestern China is characterized by an arid climate, relatively high wind speeds, and intense solar radiation. During service, concrete structures in this region are susceptible to the combined effects of intermittent rainfall, variations in environmental humidity, and strong evaporation, thereby forming a typical wetting–drying cycle environment [14]. Under this condition, the wetting stage promotes the migration of water into the concrete, whereas the drying stage induces water evaporation, drying shrinkage, and changes in pore pressure [15]. The repeated processes of water absorption and desorption can cause moisture-induced expansion and drying-shrinkage deformation within the concrete, leading to stress concentration in the interfacial transition zone and around microcracks. With an increasing number of cycles, damage gradually accumulates, resulting in pore structure deterioration, microcrack propagation, and degradation of mechanical properties [16]. Therefore, laboratory wetting–drying cycles can reproduce the repeated moisture ingress and evaporation processes experienced by concrete in the arid regions of Northwestern China, providing a controlled approach for evaluating the resistance of CGC to deterioration under alternating wetting–drying conditions.

The properties of aggregates play a critical role in determining the mechanical performance of concrete. Accordingly, strengthening CGA is an effective strategy for improving the overall performance of CGC. Sodium silicate, owing to its favorable alkali-activating and cementitious characteristics, as well as its low cost, simple operation, technical feasibility, and economic viability, has been widely considered a promising modifier for porous aggregates. Bui et al. [17] reported that recycled concrete incorporating sodium silicate-modified recycled aggregates showed increases of 33.0–50.0% in compressive strength, 33.0–41.0% in splitting tensile strength, and 15.5–42.5% in the relative dynamic modulus of elasticity compared with recycled concrete incorporating untreated recycled aggregates. Trivedi et al. [18] adopted a two-stage modification method combining mechanical abrasion and sodium silicate immersion for recycled coarse aggregates. This treatment effectively improved the mechanical properties, microstructure, and durability of recycled aggregate concrete. Yang et al. [19] demonstrated that sodium silicate modification of recycled aggregates for pervious concrete improved the ITZs between the recycled aggregates and the cementitious matrix, thereby reducing internal cracks and pores. Yin et al. [20] reported that the incorporation of silica fume and glass fibers improved the compressive and splitting tensile strengths of recycled concrete compared with plain recycled concrete, particularly in single-fiber-reinforced and hybrid-fiber-reinforced mixtures. However, when the fiber dosage exceeded 2%, both strength indices were adversely affected.

Waterborne epoxy resin exhibits strong adhesion, low curing shrinkage, corrosion resistance, and water resistance, making it a promising modifier for improving the performance of recycled aggregate concrete. Pang et al. [21] investigated the influence of waterborne epoxy resin emulsion on cement hydration and microstructural evolution and reported that the layered network structure formed by the epoxy resin effectively refined the pore structure of cement-based materials. Aggarwal [22] investigated the toughening effects of epoxy resin and polyacrylic emulsion on ordinary cement mortar and found that epoxy resin emulsion was more effective at enhancing mortar performance. Zhang et al. [23] reported that the early hydration of cement was delayed due to the formation of an epoxy resin film on the surface of cement particles. Pang et al. [24] found that waterborne epoxy resin is one of the most promising polymer modifiers owing to its excellent compatibility with cement hydration, favorable coupling effect, and superior overall performance. Shahmansouri et al. [25] further reported that biochar incorporation in high-strength engineered cementitious composites affected mechanical performance, durability-related behavior, microstructural characteristics, and the ITZ, while also offering potential environmental benefits. In another study, the incorporation of natural zeolite was shown to improve the mechanical and durability performance of concrete exposed to sodium sulfate attack, while lifecycle assessment demonstrated its potential environmental advantages [26].

However, the combined effects of chemical activation and organic film formation on the evolution of the ITZ and microstructure of coal gangue concrete during repeated wetting–drying cycles remain insufficiently understood. Furthermore, the respective roles of chemical activation and physical sealing within the combined modification system have not yet been systematically clarified. In this study, the treatment combining sodium silicate and waterborne epoxy resin is defined as a combined chemical–physical modification strategy, in which sodium silicate provides chemical activation by promoting the formation of hydration products, while waterborne epoxy resin provides physical sealing through film formation. Therefore, CGC was prepared using coal gangue coarse aggregates subjected to sodium silicate modification alone or combined chemical–physical modification using sodium silicate and waterborne epoxy resin. The evolution of its mechanical properties under wetting–drying cycles was systematically investigated. Microstructural characterization was further conducted to examine the effects of wetting–drying cycles on the micromorphology, ITZ, and pore structure of CGC. Based on these results, the damage evolution mechanisms of CGC subjected to single and combined modification were analyzed. This study provides theoretical support for the engineering application of CGC in harsh environments and offers a feasible pathway for local material utilization and green construction in Northwest China, in line with the national demand for energy conservation and emission reduction under the dual-carbon goals [27].

## 2. Materials and Methods

### 2.1. Materials

**Figure 1:**
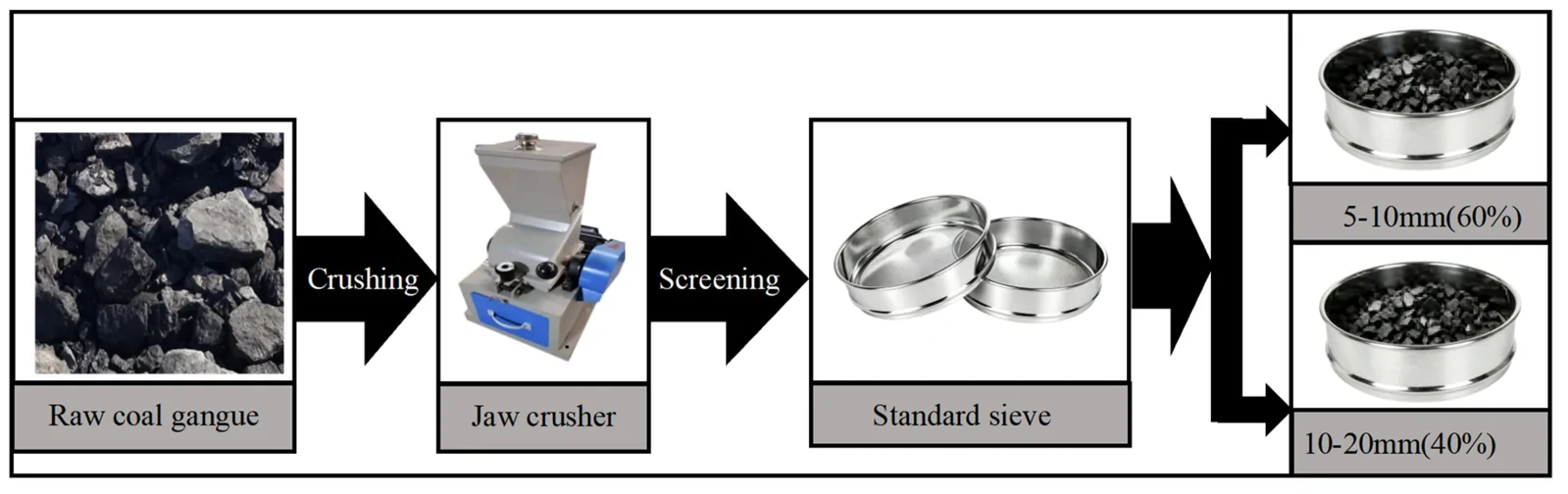
Preparation process of crushed coal gangue.

The coarse aggregates comprised natural crushed stone and coal gangue, with particle sizes ranging from 5 mm to 10 mm. The natural crushed stone, coal gangue, and natural river sand were supplied by Mengxi Cement Direct Sales Outlet (Ordos, China). The processing procedure of crushed coal gangue is illustrated in Figure 1. Natural river sand was used as the fine aggregate, and ordinary tap water was used as the mixing water. F0705 waterborne epoxy resin emulsion (Henan Jintai Chemical Co., Ltd., Zhengzhou, China), DY-175 curing agent (Henan Jintai Chemical Co., Ltd., Zhengzhou, China), and sodium silicate solution (Tianjin Chemical Reagent Co., Ltd., Tianjin, China) were used as modifiers. P.O 42.5 ordinary Portland cement (Mengxi Cement Direct Sales Outlet, Ordos, China) with a specific surface area of 369 m^2^/kg was used as the cementitious material. A powdered naphthalene-based water-reducing admixture (Henan Borun Foundry Materials Co., Ltd., Zhengzhou, China) with a water-reducing rate of 16% was used. The chemical composition data of the cement and coal gangue were obtained from quality inspection reports provided by the corresponding suppliers and are summarized in Table 1, while the physical properties of the waterborne epoxy resin emulsion and curing agent are presented in Table 2 and Table 3.

### 2.2. Experimental Methods

#### 2.2.1. Modification Test of CGA

**Figure 2:**
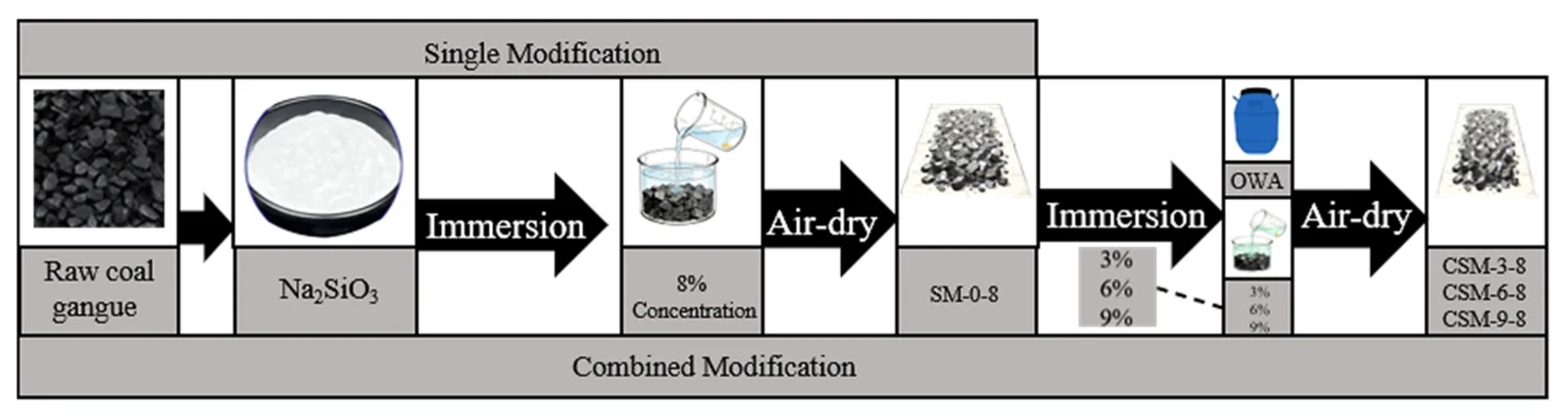
Modification process of coal gangue coarse aggregates.

Based on previous studies [28,29,30], coal gangue coarse aggregates were modified using an 8 wt.% sodium silicate solution for single modification. On this basis, aqueous waterborne epoxy modifying solutions with concentrations of 3 wt.%, 6 wt.%, and 9 wt.% were used for combined chemical–physical modification. The reported 3, 6, and 9 wt.% values refer to the mass fraction of the combined waterborne epoxy resin emulsion and curing agent relative to the total mass of the aqueous epoxy modifying solution, which consisted of the waterborne epoxy resin emulsion, curing agent, and water. The mass ratio of the waterborne epoxy resin emulsion to the curing agent was fixed at 100:85. The two components were thoroughly mixed under low-speed mechanical stirring (Guangzhou Boletai Technology Co., Ltd., Guangzhou, China) for at least 5 min until a homogeneous and stable mixture was obtained. In this study, a combined chemical–physical modification process involving sodium silicate-induced chemical activation and waterborne epoxy resin-based physical coating was adopted. For the single-modification group, the CGAs were immersed in an 8 wt.% sodium silicate solution for 5 h. For the combined-modification groups, the CGAs were first immersed in the 8 wt.% sodium silicate solution for 5 h and air-dried, followed by a second 5 h immersion in the waterborne epoxy resin-containing solution. During each immersion stage, the solution level was maintained 5–10 cm above the aggregate surface, and the solution was manually stirred every 2 h. After the final immersion treatment, the aggregates were removed, spread on plastic sheets, and air-dried in a well-ventilated area for 7 d. The modification process of the coal gangue coarse aggregates is shown in Figure 2. The specimen labels were defined according to the modification method and modifier concentration. “SM” denotes a single modification with sodium silicate, whereas “CSM” denotes combined chemical–physical modification with sodium silicate and waterborne epoxy resin. In the specimen labels, the first number represents the concentration of the aqueous epoxy modifying solution, while the second number represents the concentration of the sodium silicate solution. For example, CSM–6–8 represents combined modification using a 6 wt.% aqueous epoxy modifying solution and an 8 wt.% sodium silicate solution. The detailed modification schemes for all specimen groups are summarized in Table 4.

#### 2.2.2. Design of Mix Proportions

Based on previous studies [31,32,33] and considering the workability and mechanical performance of CGC, a 25% coal gangue coarse aggregate replacement ratio was selected. The water-to-binder ratio was approximately 0.40, and the fly ash dosage was fixed at 47 kg/m^3^, corresponding to approximately 9.85 wt.% of the total binder mass. Referring to the commonly used prism specimen size for mechanical property testing of cement-based materials [34,35], 40 mm × 40 mm × 160 mm prism specimens were prepared. For each mixture, 27 specimens were cast, and a total of six groups were designed and prepared. The detailed mix proportions are presented in Table 5.

#### 2.2.3. Wetting–Drying Cycle Test

**Figure 3:**
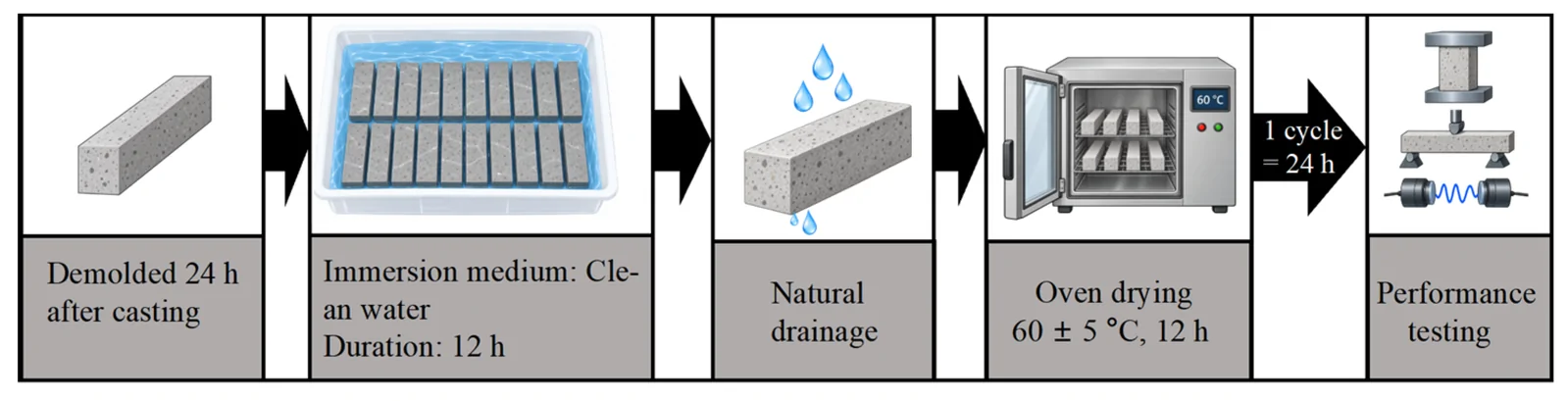
Schematic illustration of the wetting–drying cycle procedure.

Wetting–drying cycle tests were conducted using 40 mm × 40 mm × 160 mm prismatic specimens. The test regime was developed with reference to the general principles of relevant durability test methods and was adjusted according to the specimen size and the objectives of this study. The overall test procedure is shown in Figure 3. The specimens were demolded 24 h after casting and then subjected to wetting–drying cycles. Each cycle lasted 24 h and consisted of 12 h of immersion in clean water followed by 12 h of drying. During immersion, the water level was maintained at approximately 20 mm above the top surface of the specimens. After immersion, excess surface water was allowed to drain naturally, and the specimens were then directly transferred to an oven (Hanhua Intelligent Technology (Shaoxing) Co., Ltd., Shaoxing, China) and dried at 60 ± 5 °C. The immersion water temperature was not independently controlled or monitored, and the immersion stage was conducted under the ambient laboratory conditions. The laboratory temperature was maintained at 21–25 °C, with a relative humidity of 50–65%. Compressive strength, flexural strength, and relative dynamic modulus of elasticity were measured after 1, 3, 7, 14, 28, 60, 90, and 120 cycles. The selected wetting–drying regime was intended to represent the alternating moisture ingress and evaporation experienced by concrete in the arid and semi-arid regions of Northwestern China, rather than to reproduce the field climate exactly.

#### 2.2.4. Evaluation of Modified CGA Properties

(1) Twenty-Four-Hour Water Absorption

The CGA samples were first oven-dried to constant mass and then cooled to room temperature before recording their oven-dry mass using an electronic balance (Shanghai Youke Instrument Co., Ltd., Shanghai, China). Subsequently, the samples were completely immersed in clean water for 24 h. After immersion, the surface moisture was gently removed, and the saturated surface-dry mass was measured. The 24 h water absorption was calculated based on the mass change before and after immersion, as expressed in Equation (1):

(1) $$W_{24}=\frac{m_{1}-m_{0}}{m_{0}}\times 100\%$$

where $W_{24}$ is the 24 h water absorption of the specimen (%); $m_{1}$ is the saturated surface-dry mass of the specimen after 24 h of water immersion (g); and $m_{0}$ is the oven-dried mass of the specimen (g).

(2) Crushing Value

Aggregates with the specified particle size range were sampled and placed into the crushing value mold in layers, followed by leveling and standard loading using a compression testing machine (Cangzhou Honghaojieke Construction Instrument Co., Ltd., Cangzhou, China). After loading, the crushed samples were sieved through the specified sieve size, and the mass of the passing fine particles was measured. The aggregate crushing value was then calculated according to Equation (2):

(2) $$Q_{e}=\frac{m_{c}}{m}\times 100\%$$

where $Q_{e}$ is the aggregate crushing value (%); $m_{c}$ is the mass of fine particles passing through the specified sieve after compression (g); and m is the total mass of the aggregate specimen (g).

#### 2.2.5. Evaluation of the Modification Effect of CGC

(1) Compressive and Flexural Strengths

According to the Standard for Test Methods of Concrete Physical and Mechanical Properties (GB/T 50081-2019) [36], the compressive and flexural strengths of the specimens were measured using a WHY-20002 computer-controlled testing machine (Cangzhou Honghaojieke Construction Instrument Co., Ltd., Cangzhou, China). The loading rate was controlled at 0.5 MPa/s for the compressive strength test and 0.05 MPa/s for the flexural strength test. Three specimens were tested for each group, and the average value was taken as the strength result, with an accuracy of 0.1 MPa. The calculation formulas for compressive strength and flexural strength are given in Equations (3) and (4), respectively:

(3) $$F_{cu}=0.95\times \frac{F}{A}$$

where $F_{cu}$ is the compressive strength of the concrete specimen (MPa); F is the failure load of the concrete specimen (N); A is the load-bearing area of the concrete specimen (mm^2^); and 0.95 is the size conversion coefficient.

(4) $$f_{f}=\frac{3Fl}{2bh^{2}}$$

where $f_{f}$ is the flexural strength of the concrete specimen (MPa); F is the failure load of the concrete specimen (N); l is the span between the supporting rollers (mm); h is the height of the specimen cross-section (mm); and b is the width of the specimen cross-section (mm).

(2) Relative Dynamic Modulus of Elasticity

The dynamic elastic modulus of the specimens was determined by the resonance frequency method using a NELD-DTV dynamic elastic modulus tester (Xiangtan Instrument and Meter Co., Ltd., Xiangtan, China). For each group, three specimens were tested, and their average value was used as the representative result. The relative dynamic elastic modulus was calculated using Equation (5).

(5) $$E_{rd}=\frac{E_{n}}{E_{0}}=\frac{V_{n}^{2}}{V_{0}^{2}}$$

where $E_{rd}$ is the relative dynamic modulus of elasticity of concrete after n wetting–drying cycles; $E_{0}$ is the initial dynamic modulus of elasticity of concrete (MPa); $E_{n}$ is the dynamic modulus of elasticity of concrete after n wetting–drying cycles (MPa); $V_{n}$ is the transverse fundamental frequency of concrete after n wetting–drying cycle (Hz); and $V_{0}$ is the initial transverse fundamental frequency of concrete before wetting–drying cycling (Hz).

#### 2.2.6. XRD

Powder samples were prepared by drilling and grinding the core region of the specimens, followed by sieving them through a 75 μm sieve. XRD analysis was performed using a Bruker D8 Advance diffractometer (Bruker AXS GmbH, Karlsruhe, Germany) over a scanning range of 5–70°, with a step size of 0.02626°. The obtained XRD patterns were analyzed using Jade software (version 6.5, Materials Data, Inc., Livermore, CA, USA).

#### 2.2.7. SEM-EDS

**Figure 4:**
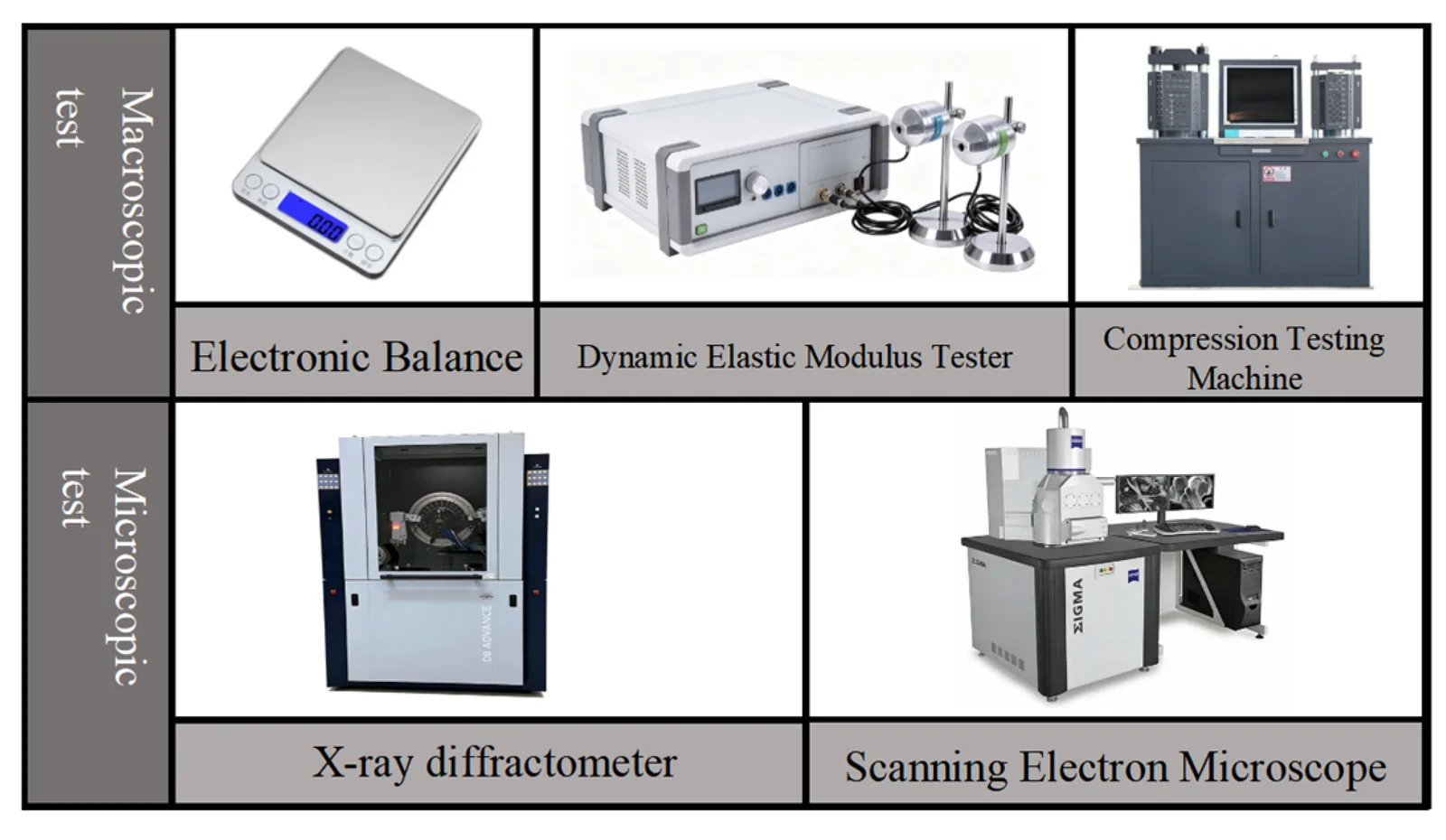
Test procedure and experimental equipment.

The microstructural morphology and ITZ characteristics of CGC were observed using SEM, and the elemental composition of unidentified structures was determined by EDS. Both analyses were performed using the same SEM–EDS system (Beijing Purui Jingsaisi Instrument Co., Ltd., Beijing, China). The main instruments and equipment used in the experiments are shown in Figure 4.

#### 2.2.8. Estimation of Carbon Emission Reduction

Based on previous lifecycle assessment studies on concrete [37,38], the carbon emission reduction resulting from the replacement of natural coarse aggregate with coal gangue coarse aggregate per unit volume of concrete was estimated. The carbon emission factor for the production stage of natural coarse aggregate was taken as $EF_{NA}=0.004\;\mathrm{kg}\;{\mathrm{CO}}_{2}\text{-}\mathrm{eq}/\mathrm{kg}$. Since coal gangue is an industrial solid waste, only the carbon emissions generated during crushing and pretreatment were considered, with the corresponding emission factor set as [39]. The carbon emission reduction from natural aggregate replacement was calculated according to Equation (6):

(6) $$\Delta C_{1}=m_{NA}\cdot EF_{NA}-m_{CGA}\cdot EF_{CGA}$$

where $\Delta C_{1}$ is the carbon emission reduction per unit volume of concrete in kg CO_2_-eq/m^3^; $m_{\mathrm{NA}}$ is the reduced mass of natural coarse aggregate in kg/m^3^; $m_{\mathrm{CGA}}$ is the introduced mass of coal gangue coarse aggregate in kg/m^3^; $EF_{\mathrm{NA}}$ is the emission factor of natural coarse aggregate production in kg CO_2_-eq/kg; and $EF_{\mathrm{CGA}}$ is the emission factor of coal gangue coarse aggregate crushing and pretreatment in kg CO_2_-eq/kg.

## 3. Results

### 3.1. Effect of Modification Methods on CGA

**Figure 5:**
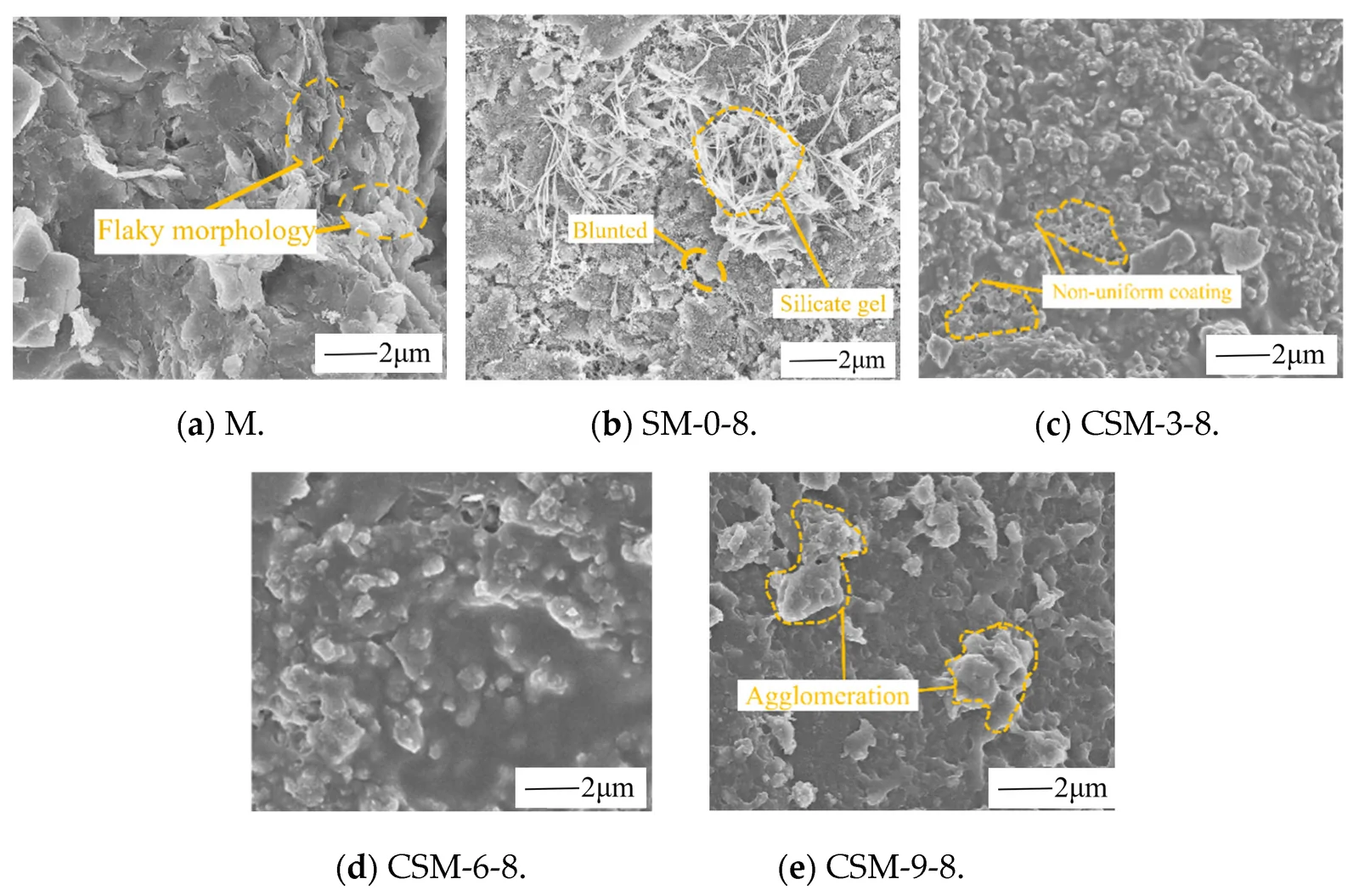
SEM images of modified CGA.

The microscopic morphologies of CGA modified by different methods are shown in Figure 5. As shown in Figure 5a, the surface of M exhibits a flake-like morphology with sharp edges, accompanied by numerous pores, microcracks, and loose particles, indicating a relatively loose and defective structure. This morphology is one of the main reasons for the high water absorption and low strength of CGA. As shown in Figure 5b, compared with M, the corners and sharp edges on the surface of SM–0–8 are noticeably smoothed. This improvement may be associated with interactions between sodium silicate and potentially reactive Si- and Al-bearing phases on the coal gangue surface under alkaline conditions, which may promote the formation of silicate gels or gel-like deposits on the aggregate surface. These products partially fill and seal the original pores and cracks, thereby improving the surface compactness of the aggregates. However, due to the limited modification effect, some microcracks still remain inside and on the surface of the aggregates, which may provide pathways for moisture and aggressive ions and thus adversely affect the long-term durability of concrete.

Under combined chemical–physical modification, the CGA surface exhibited a relatively continuous coating-like morphology, which was interpreted as being associated with the cured epoxy resin and may have contributed to the reduction in visible surface pores and cracks. The dosage of waterborne epoxy resin had a pronounced influence on the modification effect. As shown in Figure 5c, when the resin dosage was 3 wt.%, the lower resin dosage may have been insufficient to develop a relatively continuous coating on the surface of the CGA. As a result, obvious cracks and pores were still observed, together with locally exposed areas. As shown in Figure 5d, when the resin dosage was 6 wt.%, a relatively continuous and dense coating-like layer was observed on the aggregate surface, which may have been associated with the cured epoxy resin and may have contributed to the reduction of visible surface pores and microcracks as well as the improvement of aggregate integrity. As shown in Figure 5e, when the resin dosage was increased to 9 wt.%, the higher resin dosage may have promoted local agglomeration during curing, resulting in uneven film thickness on the aggregate surface. This may adversely affect the macroscopic properties of concrete.

In summary, incorporating waterborne epoxy resin into the sodium silicate modification process resulted in a superior overall modification effect compared with sodium silicate treatment alone. Waterborne epoxy resin partially improved the loose and porous surface structure of CGA. However, the modification effect was strongly dependent on the resin dosage. At a waterborne epoxy resin dosage of 6 wt.%, the aggregate surface exhibited the most complete coating among the tested dosages, and the overall structure became denser, indicating the best modification effect within the investigated dosage range. This observation is generally consistent with the subsequent physical property test results. Similar improvements in aggregate surface morphology were also reported by Wang et al. [30], who found that composite modification reduced surface defects and improved the interfacial characteristics of coal gangue aggregate concrete.

### 3.2. Physical Property Analysis of Modified CGA

#### 3.2.1. Twenty-Four-Hour Water Absorption

**Figure 6:**
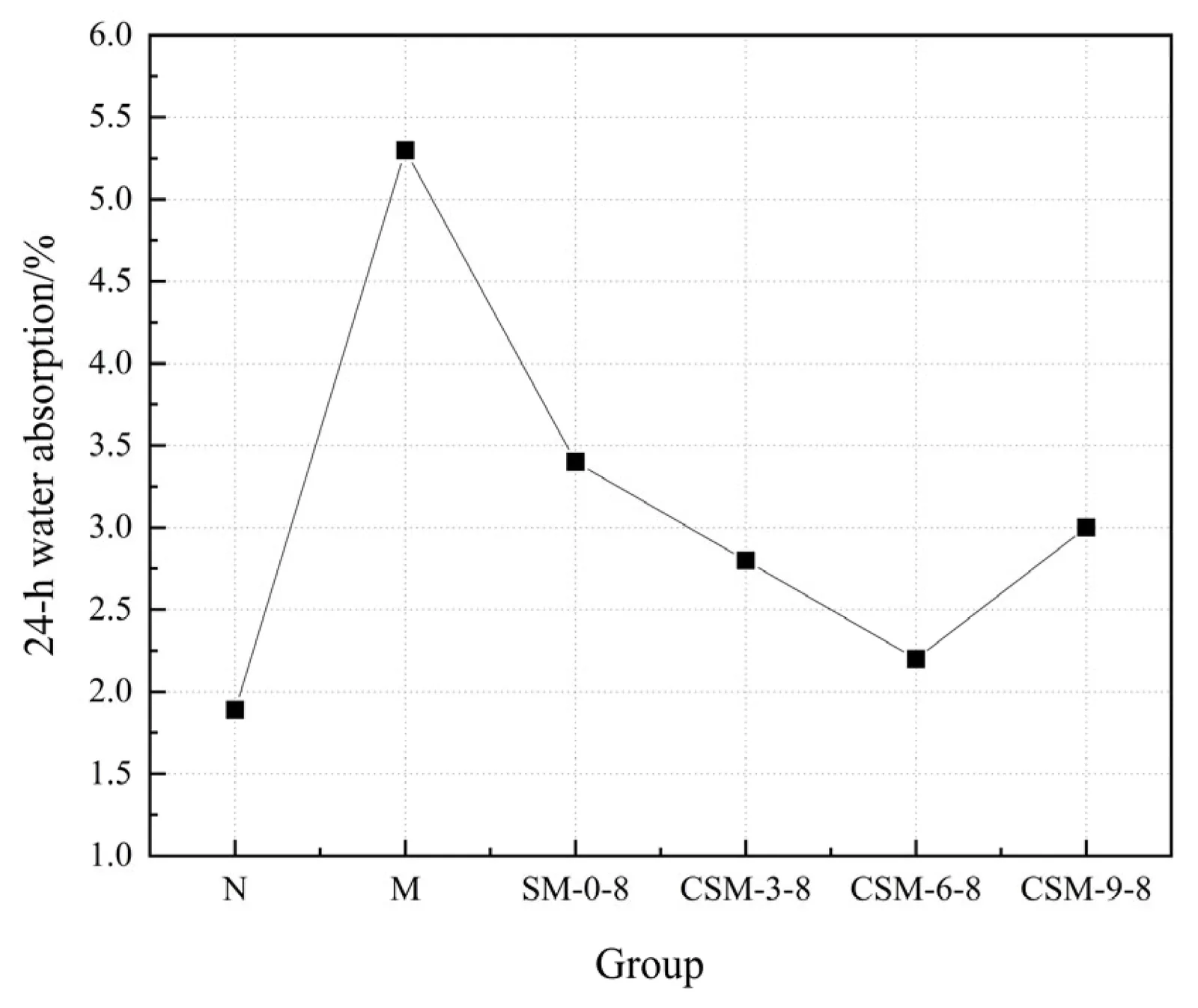
Twenty-four-hour water absorption of CGA.

The 24 h water absorption of the CGA is shown in Figure 6. Among all groups, M exhibits the highest water absorption, which can be attributed to the inherently loose and porous structure of the unmodified coal gangue surface. Such high water absorption may adversely affect the durability of CGA.

The water absorption of SM–0–8 was lower than that of M. This reduction may be associated with gel-like products formed during sodium silicate modification, which may contribute to partially filling pores and cracks on the aggregate surface, improving aggregate compactness, and reducing potential water ingress pathways. Compared with SM–0–8, CSM–3–8, CSM–6–8, and CSM–9–8 exhibited further reductions in water absorption. This reduction may be associated with an epoxy-related surface coating formed after the incorporation of waterborne epoxy resin, which may have contributed to further reducing the accessibility of residual pores remaining after sodium silicate modification. Consequently, the aggregate structure became denser and more integrated, thereby more effectively preventing water penetration. For the combined chemical–physical modification groups, water absorption showed a clear dependence on the dosage of waterborne epoxy resin. As the resin dosage increased from 3 wt.% to 6 wt.%, water absorption continued to decrease, whereas a slight increase was observed when the dosage was further increased to 9 wt.%. This indicates that, among the investigated resin dosages, the 6 wt.% group exhibited a more complete surface coating and the lowest water absorption, suggesting comparatively improved aggregate compactness and resistance to water ingress within the tested dosage range. A similar reduction in water absorption was reported by Geng et al. [40], who found that epoxy-resin surface treatment effectively reduced the water absorption of recycled coarse aggregates.

#### 3.2.2. Crushing Value of CGA

**Figure 7:**
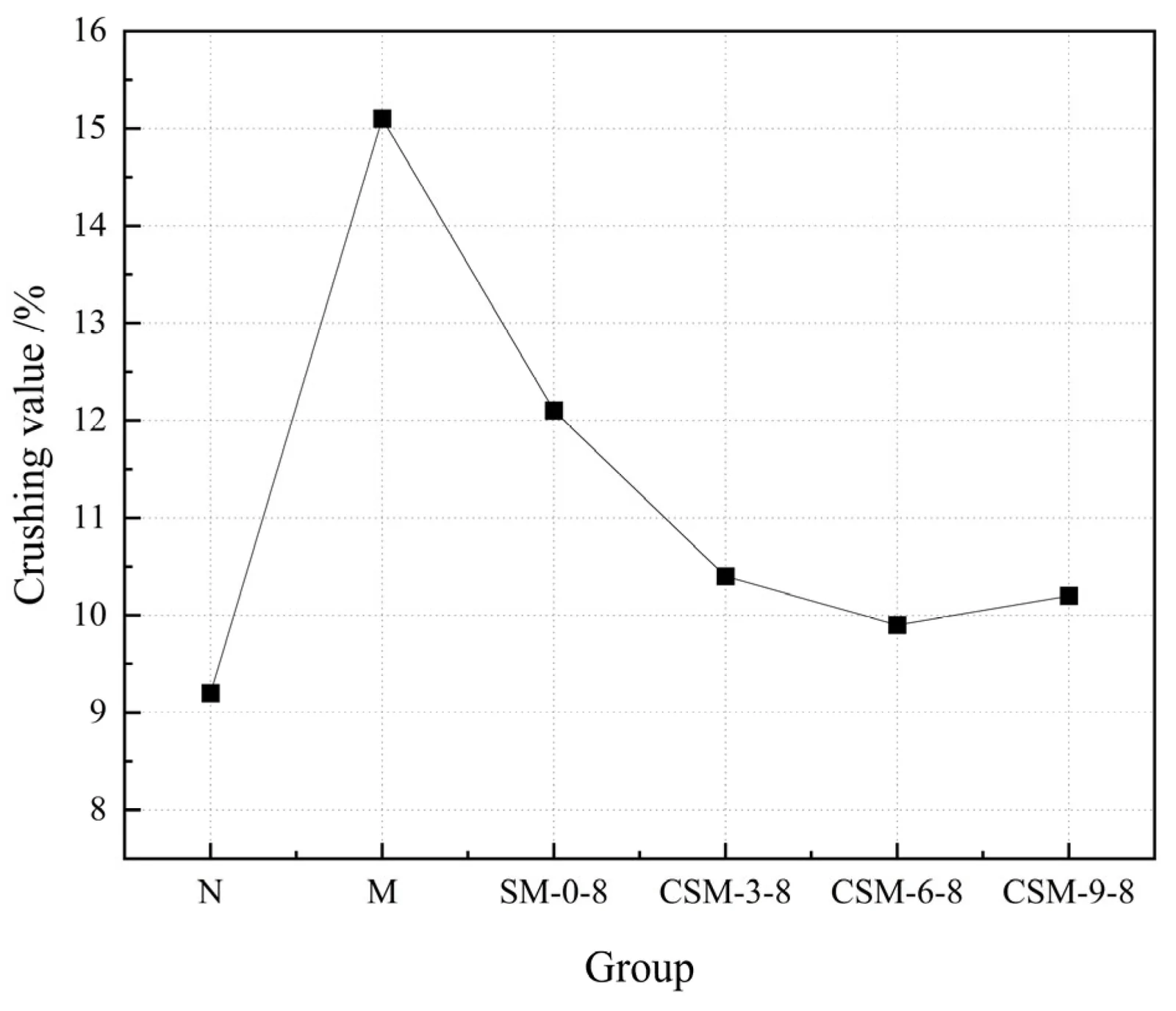
Crushing value of modified CGA.

The crushing value of CGA is shown in Figure 7. M exhibits the highest crushing value, indicating that the coal gangue itself has relatively low strength and is more prone to crushing under external loading. This result can be attributed to the presence of numerous pores and microcracks within and on the surface of the coal gangue. These defects act as stress concentration sites, thereby reducing the crushing resistance of the aggregates.

The crushing values of the CGA varied markedly under different modification treatments. Compared with M, SM–0–8, CSM–3–8, CSM–6–8, and CSM–9–8 all exhibited lower crushing values, indicating that the modification treatments effectively improved the resistance of CGA to damage under external loading. Moreover, CSM–3–8, CSM–6–8, and CSM–9–8 all exhibited lower crushing values than SM–0–8, indicating that the incorporation of waterborne epoxy resin in combination with sodium silicate provided an additional improvement in the crushing resistance of CGA. Combined chemical–physical modification further enhanced the internal structural stability of the aggregates and improved their overall particle strength. Among the combined chemical–physical modification groups, CSM–6–8 exhibited the lowest crushing value, indicating the strongest resistance to crushing. When the waterborne epoxy resin dosage was 3 wt.%, the lower resin dosage may have been insufficient to develop a relatively continuous coating on the surface of the CGA. In contrast, when the dosage was increased to 9 wt.%, the higher resin dosage may have promoted local resin agglomeration, resulting in uneven coating thickness and stress distribution on the aggregate surface, which consequently weakened the crushing resistance of the aggregates.

### 3.3. Mechanical Properties of Concrete

#### 3.3.1. Analysis of Compressive and Flexural Strengths

**Figure 8:**
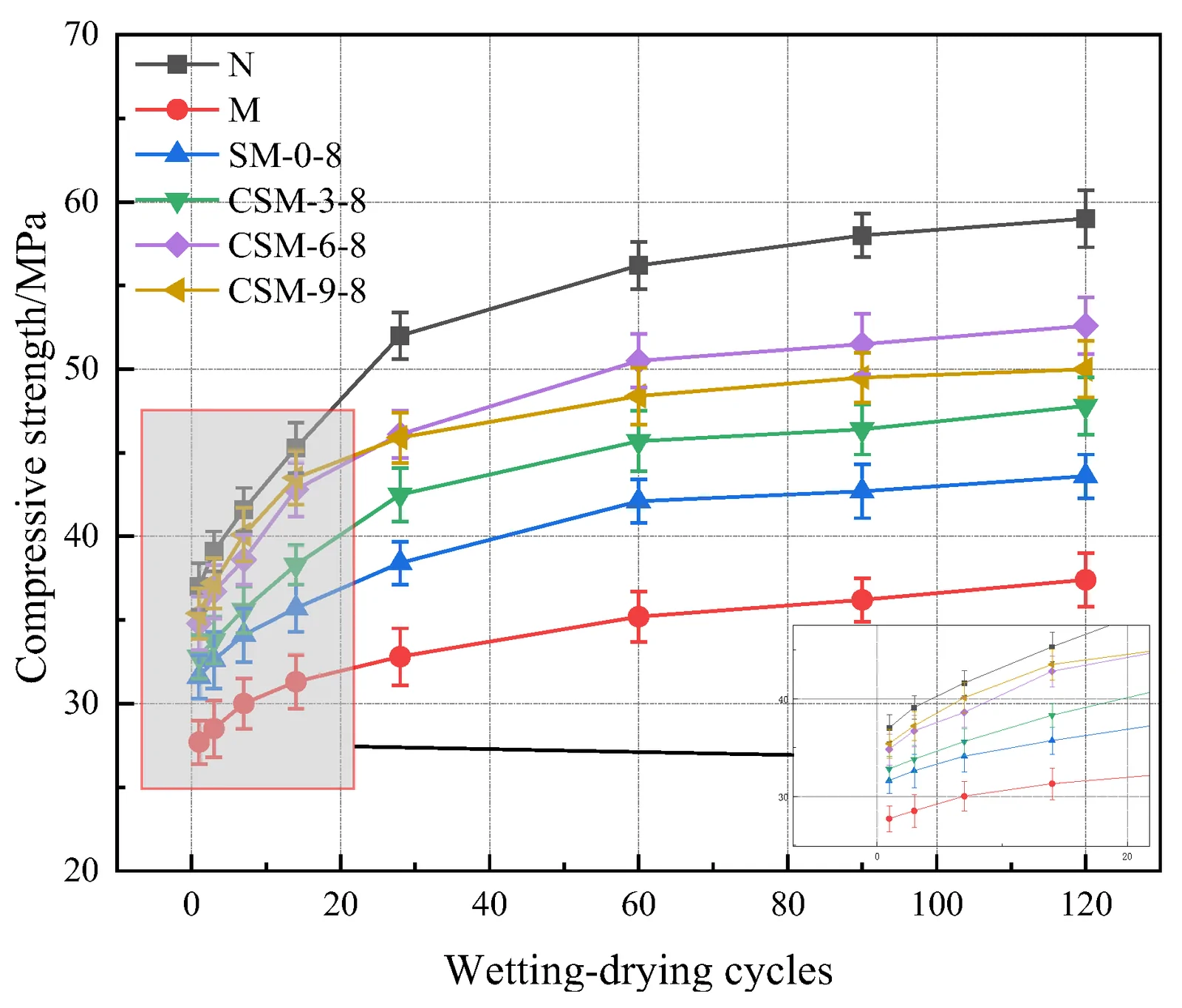
Variation in compressive strength of concrete under wetting–drying cycles.

**Figure 9:**
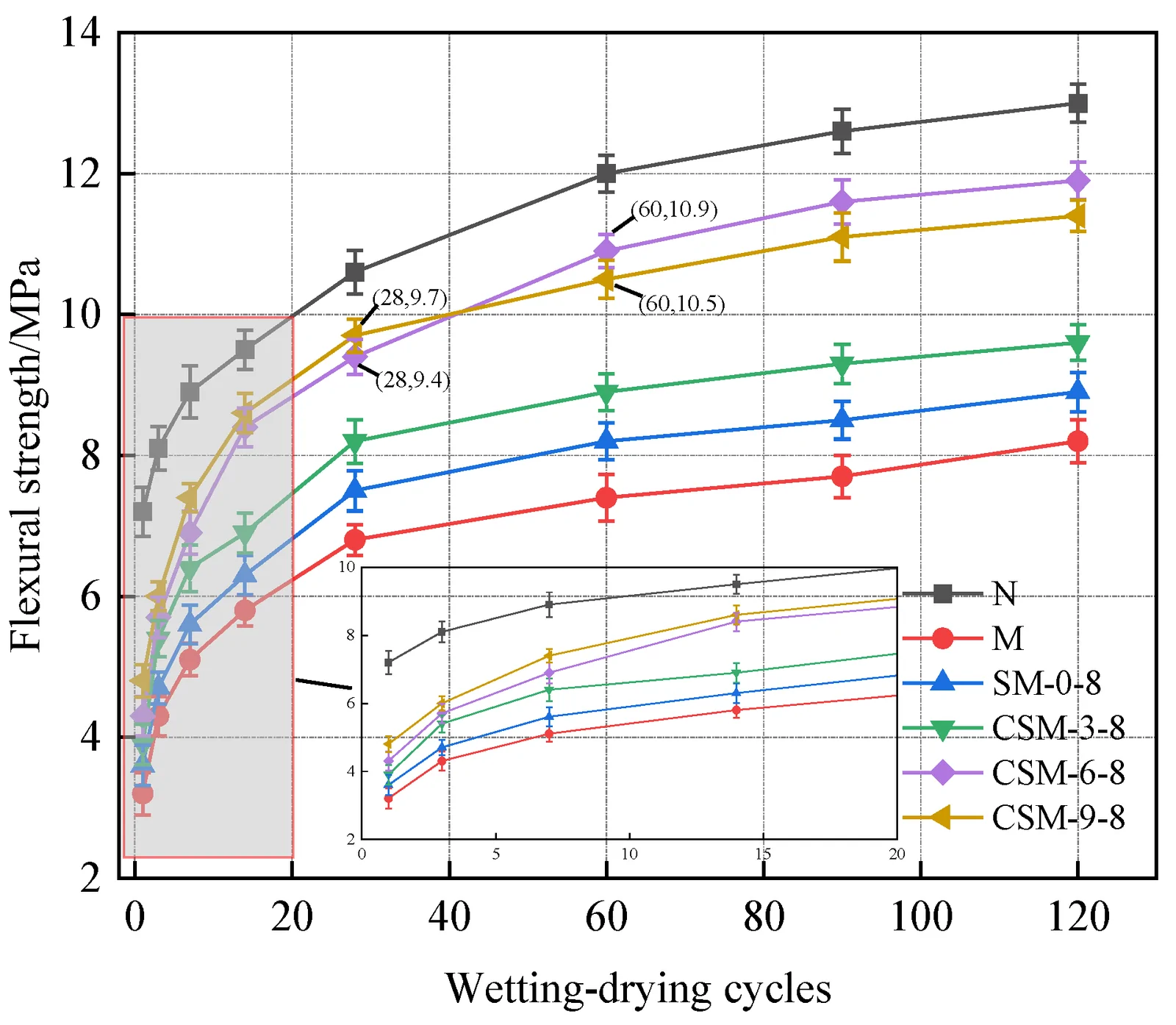
Variation in flexural strength of concrete under wetting–drying cycles.

The compressive and flexural strengths of the specimens are shown in Figure 8 and Figure 9, respectively. After different numbers of wetting–drying cycles, SM–0–8, CSM–3–8, CSM–6–8, and CSM–9–8 all exhibited higher compressive and flexural strengths than M. With increasing cycle number, both compressive and flexural strengths increased during the first 28 cycles and then gradually leveled off. The strength development during the initial 28-cycle period may be associated with several concurrent processes, including continued cement hydration, age-related material development, and the ongoing effects of the modification treatments during wetting–drying exposure.

After 28 wetting–drying cycles, SM–0–8, CSM–3–8, CSM–6–8, and CSM–9–8 exhibited compressive strengths of 38.4, 42.5, 46.1, and 45.9 MPa, respectively, representing increases of 17.07%, 29.57%, 40.54%, and 39.94% compared with M. The corresponding flexural strengths were 7.5, 8.2, 9.7, and 9.4 MPa, respectively, representing increases of 10.29%, 20.49%, 42.65%, and 38.24% relative to M. These results indicate that the modification treatments improved both the compressive and flexural strengths of CGC. A similar improvement in mechanical performance was reported by Gao et al. [41], who found that the incorporation of 1.5% nano-silica increased the 28-day compressive strength of coal gangue concrete by 14.07%.

The strength development observed during the initial 28-cycle period may result from several concurrent processes. Continued cement hydration and age-related matrix densification can contribute to the development of mechanical properties. Meanwhile, during wetting–drying exposure, sodium silicate may penetrate the pores of coal gangue and the ITZ. During the subsequent drying stage, the sodium silicate solution becomes concentrated, which may promote interactions with potentially reactive Si- and Al-bearing phases in the coal gangue, thereby contributing to the formation of additional gel products. These processes may further improve the compactness of the matrix and the aggregate–paste interface.

After 60 wetting–drying cycles, CSM–3–8, CSM–6–8, and CSM–9–8 exhibited compressive strengths of 45.7, 50.5, and 48.4 MPa, respectively, corresponding to increases of 8.55%, 19.95%, and 14.96% compared with SM–0–8. Their corresponding flexural strengths were 8.9, 10.9, and 10.5 MPa, respectively, representing increases of 8.50%, 32.93%, and 28.04% relative to SM–0–8. These results indicate that, during the later stage of wetting–drying cycles, the flexural strength enhancement in the combined chemical–physical modification groups was more pronounced than that in the single sodium silicate modification group.

The observed behavior may be associated with the possible contribution of the resin-related coating during stress development under wetting–drying cycles. Once microcracks have initiated, the coating may dissipate part of the fracture energy through viscoelastic deformation, thereby potentially delaying crack propagation. This possible mechanism may partly explain the more pronounced improvement in flexural strength observed in the combined-modification groups. When the wetting–drying cycles were further extended to 90 cycles, the pores were gradually filled as the reaction proceeded. Meanwhile, the active components on the coal gangue surface were progressively consumed, leading to a decrease in the reaction rate. Consequently, the strength growth curve gradually tended to level off. After 120 wetting–drying cycles, the compressive and flexural strengths of CSM–6–8 remained 11.92% and 33.70% higher than those of SM–0–8, respectively, indicating that the combined modification retained a clear performance advantage during prolonged wetting–drying exposure.

Notably, after 28 wetting–drying cycles, both the compressive and flexural strengths of CSM–6–8 were higher than those of CSM–9–8. This result may be associated with the 6 wt.% waterborne epoxy resin dosage, for which the microstructural observations suggested a relatively uniform coating morphology that may have contributed to improving the pore structure of the cement matrix and the aggregate–paste interface. However, when the dosage was increased to 9 wt.%, excessive polymer particles may have locally agglomerated, preventing the formation of a completely uniform film. Under repeated shrinkage stresses induced by wetting–drying cycles, stress concentration was likely to occur around the polymer agglomerates and at their interfaces with the matrix, thereby promoting the formation of microcracks. This indicates that the modification effect of CGC did not increase monotonically with the dosage of waterborne epoxy resin. Among the three investigated dosage levels, 6 wt.% exhibited the best overall modification performance.

#### 3.3.2. Relative Dynamic Modulus of Elasticity

**Figure 10:**
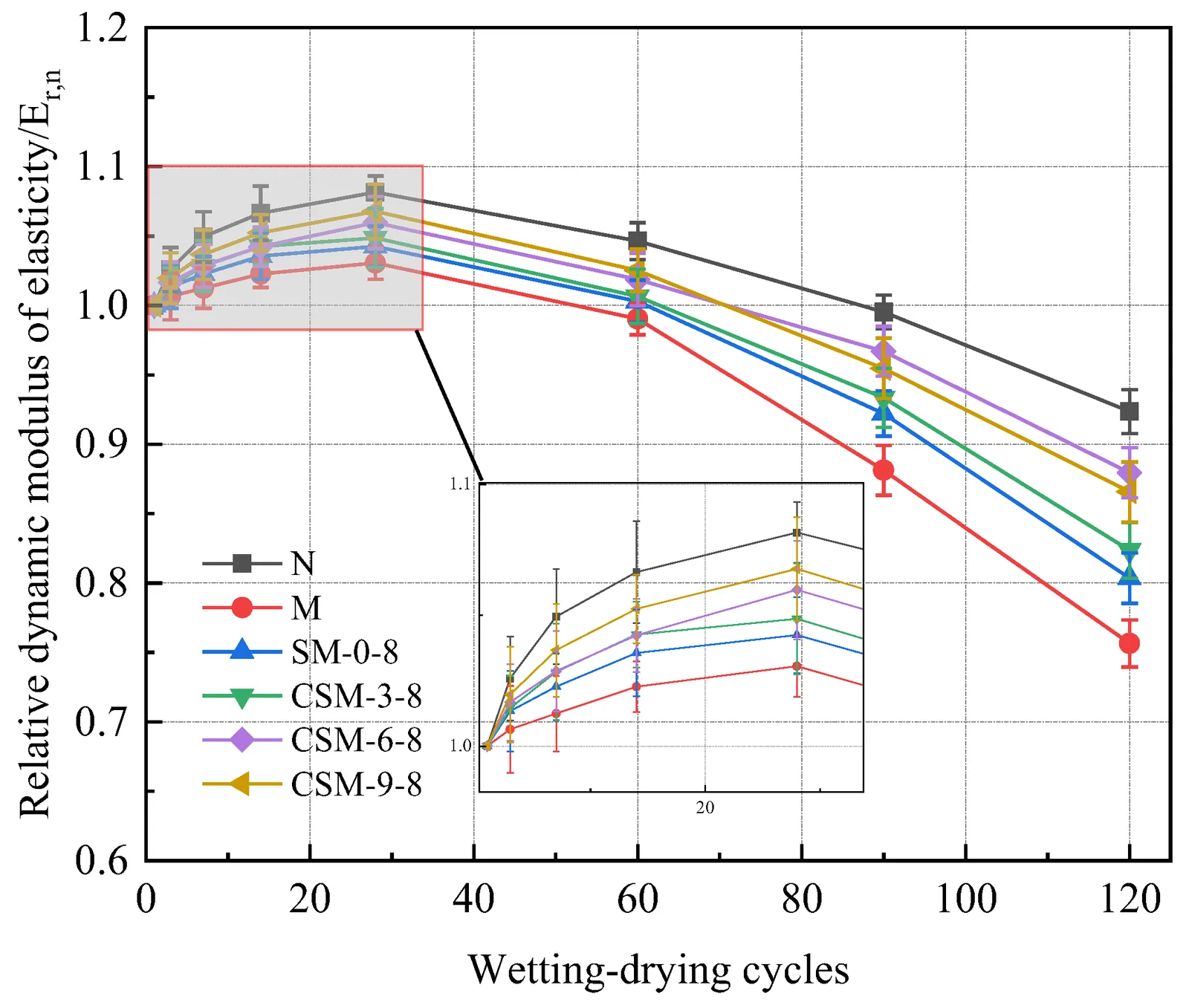
Variation in the relative dynamic modulus of elasticity with the number of wetting–drying cycles.

The variation in the relative dynamic modulus of elasticity of each group under wetting–drying cycles is presented in Figure 10. During the first 14 cycles, the relative dynamic modulus of elasticity of all specimens increased, while the rate of increase gradually slowed from 14 to 28 cycles; the maximum values were reached at 28 cycles. At this point, the relative dynamic modulus of elasticity values of SM–0–8, CSM–3–8, CSM–6–8, and CSM–9–8 were 1.03, 1.04, 1.05, and 1.06, respectively. Although CSM–9–8 exhibited the highest relative dynamic modulus of elasticity at 28 cycles, this value represents the maximum of this individual property only. Considering the overall mechanical performance and resistance to deterioration under wetting–drying cycles, CSM–6–8 exhibited the best overall performance among the investigated mixtures.

This behavior may be related to the progressive densification of the internal structure. The products generated by sodium silicate modification can fill internal pores, while, in the CSM groups, the resin film may provide an additional contribution by sealing residual defects and improving structural integrity. After 28 wetting–drying cycles, the relative dynamic modulus of elasticity of SM–0–8, CSM–3–8, CSM–6–8, and CSM–9–8 reached their respective maximum values.

Throughout the wetting–drying cycles, the relative dynamic modulus of elasticity of CSM–3–8, CSM–6–8, and CSM–9–8 remained higher than that of SM–0–8. This can be attributed to the higher compactness and lower porosity of the modified CGC compared with the unmodified counterpart. Among the CGC subjected to combined modification specimens, CSM–9–8 exhibited a higher relative dynamic modulus of elasticity than SM–0–8, CSM–3–8, and CSM–6–8 at the initial stage. However, after 60 cycles, the relative dynamic modulus of elasticity of CSM–6–8 surpassed that of CSM–9–8. After 90 wetting–drying cycles, the relative dynamic modulus of elasticity of SM–0–8, CSM–3–8, CSM–6–8, and CSM–9–8 decreased by 10.0%, 6.7%, 3.4%, and 4.6%, respectively, relative to their respective peak values at 28 cycles. This trend is generally consistent with the variations in compressive and flexural strengths, indicating that the relative dynamic modulus of elasticity can effectively reflect the damage evolution of the internal structure of CGC.

### 3.4. XRD Analysis

**Figure 11:**
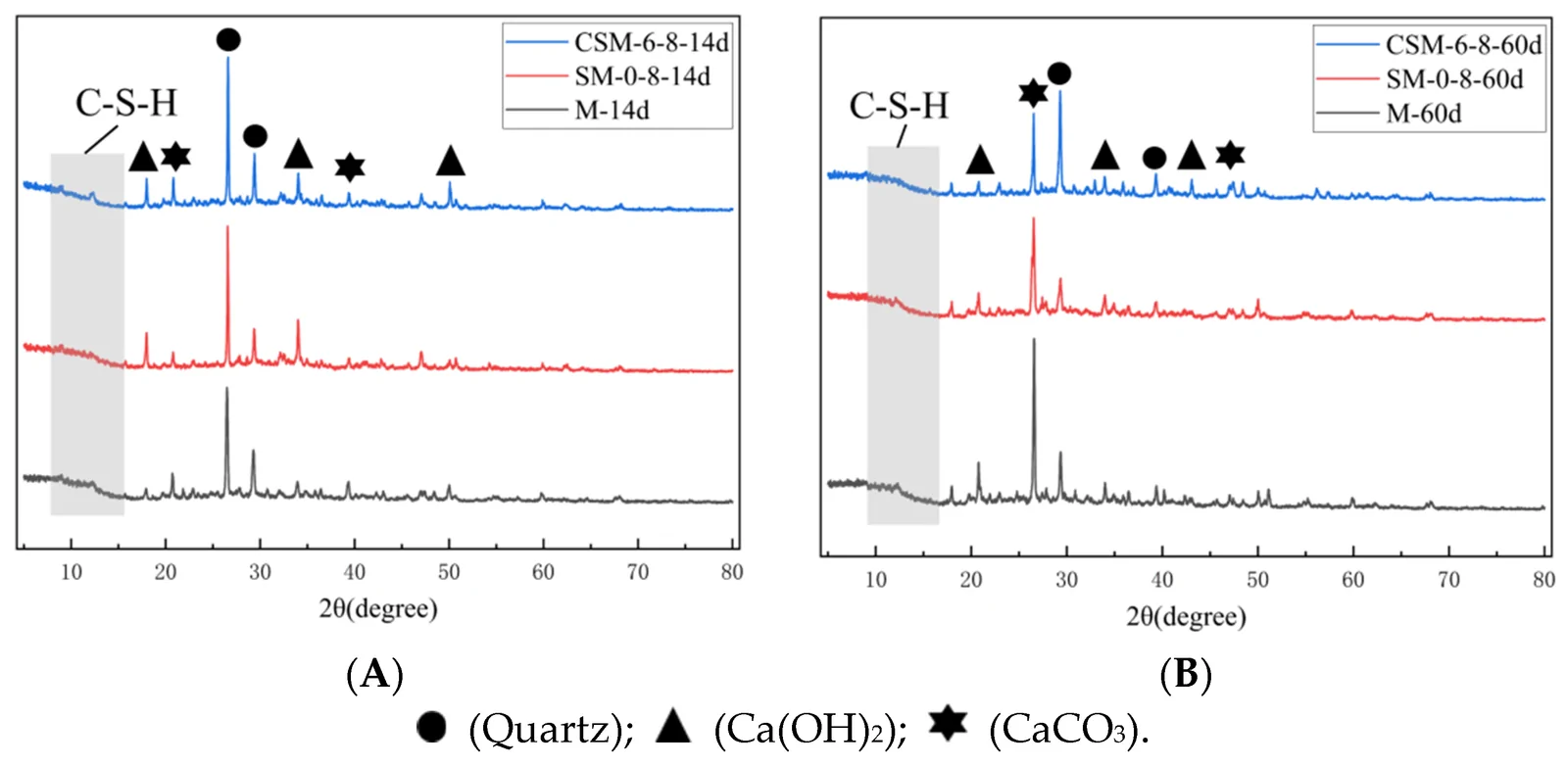
XRD patterns showing the phase evolution of modified CGC under wetting–drying cycles.

The XRD patterns of the CGC specimens after 14 and 60 wetting–drying cycles are shown in Figure 11A and Figure 11B, respectively. The main crystalline phases identified in the three groups are quartz, Ca(OH)_2_, and CaCO_3_. Compared with the unmodified group M, the diffraction peak intensities of quartz and Ca(OH)_2_ were lower in the SM–0–8 and CSM–6–8 groups. This variation may be associated with sodium silicate-related reactions under the alkaline conditions of the system, as described in Equation (7), and, together with the corresponding microstructural observations, provides supporting evidence for the proposed reaction pathway. Under these alkaline conditions, potentially reactive Si- and Al-bearing phases in coal gangue may undergo partial dissolution, as described by Equations (8) and (9). The dissolved Si- and Al-containing species may subsequently participate in cement hydration and pozzolanic reactions, thereby contributing to the formation of additional gel-like hydration products and the partial consumption of Ca(OH)_2_, as described in Equation (10). Moreover, the CSM–6–8 group still exhibits pronounced chemical activation characteristics, suggesting that the introduction of waterborne epoxy resin does not appear to hinder the sodium silicate-induced reactions involving Si- and Al-containing phases in coal gangue.

(7) $$Na_{2}SiO_{3}+(n+1)H_{2}O\to 2Na^{+}+2OH^{-}+SiO_{2}\cdot nH_{2}O$$

(8) $$SiO_{2}+2OH^{-}\to SiO_{3}^{2-}+H_{2}O$$

(9) $$Al_{2}O_{3}+2OH^{-}+3H_{2}O\to 2{\left[Al{\left(OH\right)}_{4}\right]}^{-}$$

(10) $$Ca{\left(OH\right)}_{2}+SiO_{2}+(n-1)H_{2}O\to C-S-H$$

As shown in Figure 11A,B, during the wetting–drying cycles, the XRD patterns of the SM–0–8 and CSM–6–8 groups exhibit a certain degree of background elevation. This phenomenon may reflect an increased contribution from low-crystalline or amorphous reaction products and, together with the SEM-EDS observations, supports the interpretation of possible gel-like hydration products such as C-S-H/C-A-S-H. Low-crystalline or amorphous reaction products generally do not produce sharp diffraction peaks and may instead contribute to diffuse scattering or elevated background intensity in the XRD patterns. If such low-crystalline or amorphous reaction products are formed, they may contribute to the partial filling of surface pores in the CGA and microdefects within the cementitious matrix, thereby potentially improving the interfacial transition zone and the overall compactness of the specimens.

As shown in Figure 11B, after 60 wetting–drying cycles, the diffraction peaks of Ca(OH)_2_ in all three groups weakened, whereas those of CaCO_3_ became relatively more pronounced. This phenomenon may be associated with the dissolution, migration, and carbonation of Ca(OH)_2_ during the wetting–drying process. Under repeated water absorption and drying, moisture migration inside the concrete is intensified. Meanwhile, atmospheric CO_2_ can diffuse into the interior through pores and microcracks and react with alkaline hydration products, such as Ca(OH)_2_, to form CaCO_3_. The unmodified group M contains more pores in the CGA and more developed internal moisture transport channels [42]. Therefore, the more pronounced decrease in Ca(OH)_2_ diffraction intensity and increase in CaCO_3_-related diffraction features in this group are consistent with more evident changes in the crystalline-phase characteristics and possible internal structural deterioration. In contrast, for SM–0–8, sodium silicate modification may promote reactions associated with the formation of additional gel-like hydration products, which may contribute to improved matrix compactness and reduced phase evolution to some extent. CSM–6–8 exhibited relatively stable diffraction characteristics, which are consistent with the possible combined effects of sodium silicate-related reaction products and an epoxy-associated surface coating on microstructural stability. These effects may contribute to reducing the accessibility of surface pores, limiting moisture migration, and improving the interfacial condition between the aggregates and cement paste [43]. Therefore, the group subjected to combined modification can still maintain a relatively stable internal structure during the later stages of wetting–drying cycles.

### 3.5. SEM-EDS Analysis

#### 3.5.1. Analysis of Hydration Products

**Figure 12:**
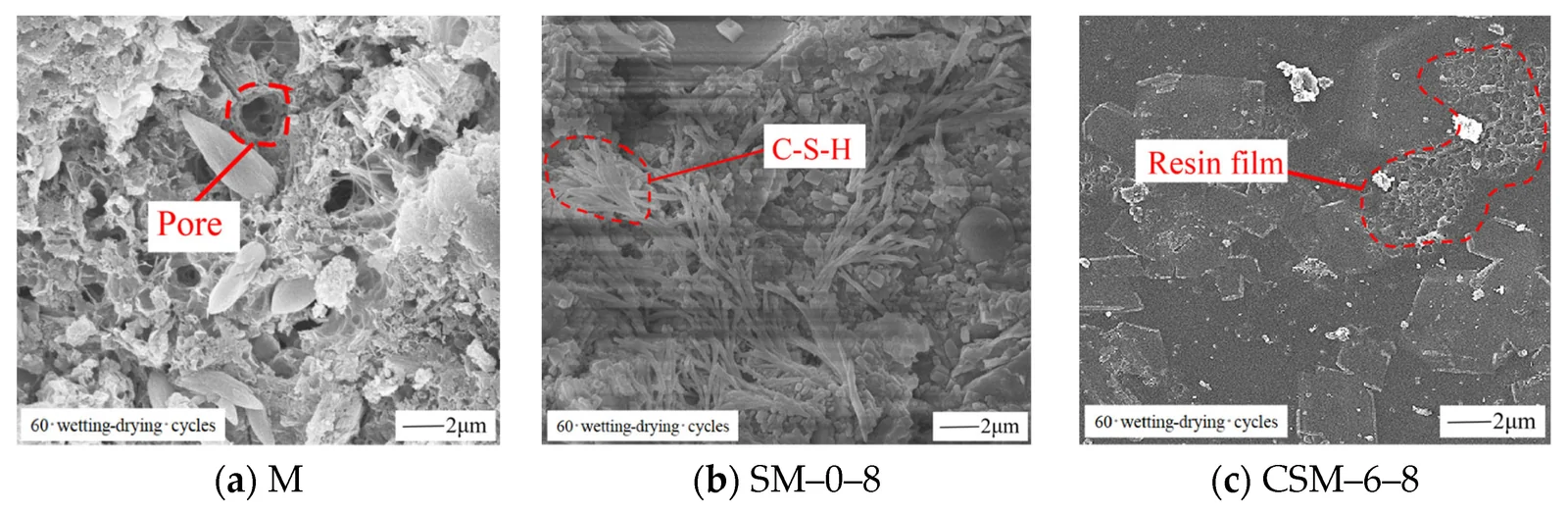
SEM images showing the microstructural characteristics of CGC subjected to single sodium silicate modification and combined chemical–physical modification under wetting–drying cycles.

**Figure 13:**
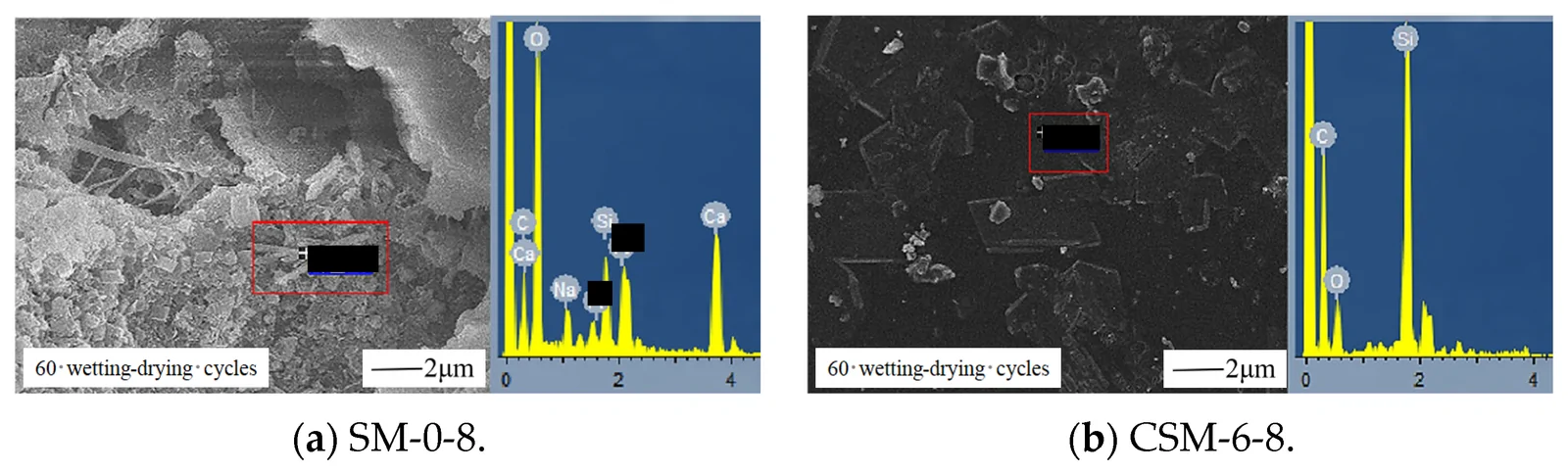
SEM-EDS characteristics of representative gel-like products and film-like regions in modified CGC under wetting–drying cycles.

As shown in Figure 12, distinct differences in microstructural morphology were observed among M, SM–0–8, and CSM–6–8 after wetting–drying cycles. The M specimen exhibited a relatively loose microstructure with visible pores and microcracks, whereas the microstructure of SM–0–8 became denser after sodium silicate modification. For CSM–6–8, the matrix and ITZ exhibited further improved compactness, with fewer visible defects. To further identify the characteristic products and surface phases observed in the modified specimens, representative regions of SM–0–8 and CSM–6–8 were analyzed by EDS, as shown in Figure 13.

According to the EDS results, the characteristic peaks of Ca, O, and Si in this region were relatively pronounced, as shown in Figure 13a. Combined with the observed microstructural morphology, the local Ca-, Si-, and O-rich elemental signature is consistent with the possible presence of C-S-H-type gel and, together with the XRD observations, provides complementary evidence for hydration-related reaction products. Under wetting–drying cycles, sodium silicate in SM–0–8 may undergo hydrolysis and release OH^−^, thereby contributing to the formation of an alkaline environment. This alkaline environment may promote the disruption of Si-O-Si and Si-O-Al bonds in potentially reactive Si- and Al-bearing phases on the coal gangue surface, thereby facilitating their participation in subsequent pozzolanic reactions [44]. Taken together, these observations support the interpretation that C-S-H- and C-A-S-H-type gel products may have formed during the modification process. The bundle-like morphology observed on the CGA surface, together with the corresponding EDS characteristics and XRD results, further supports the proposed hydration-related modification mechanism.

As shown in Figure 13b, a thin film-like morphology was observed on the surface of the CSM–6–8 specimen. Combined with the EDS results, the characteristic peaks of C, O, and Si in this region were relatively pronounced, and their corresponding peak areas were comparatively large. Therefore, the combined SEM morphology and elemental characteristics of this region are consistent with the possible presence of an epoxy-associated organic coating. During wetting–drying cycles, moisture-induced stresses and drying-shrinkage stresses were generated within the concrete, and the accumulated stresses gradually concentrated in the ITZ as the number of cycles increased, thereby impairing the structural stability of CGC [45]. The gel-like products associated with sodium silicate modification and the epoxy-associated surface coating may have played complementary roles through chemical and physical mechanisms, respectively. Specifically, the possible gel-like reaction products may have contributed to pore filling and matrix densification, whereas the epoxy-associated coating may have provided an additional sealing effect that reduced external ingress and contributed to structural stability. Together, these two effects may have contributed to pore filling and reduced external ingress, thereby providing a possible additional improvement compared with sodium silicate modification alone.

#### 3.5.2. ITZ Analysis

**Figure 14:**
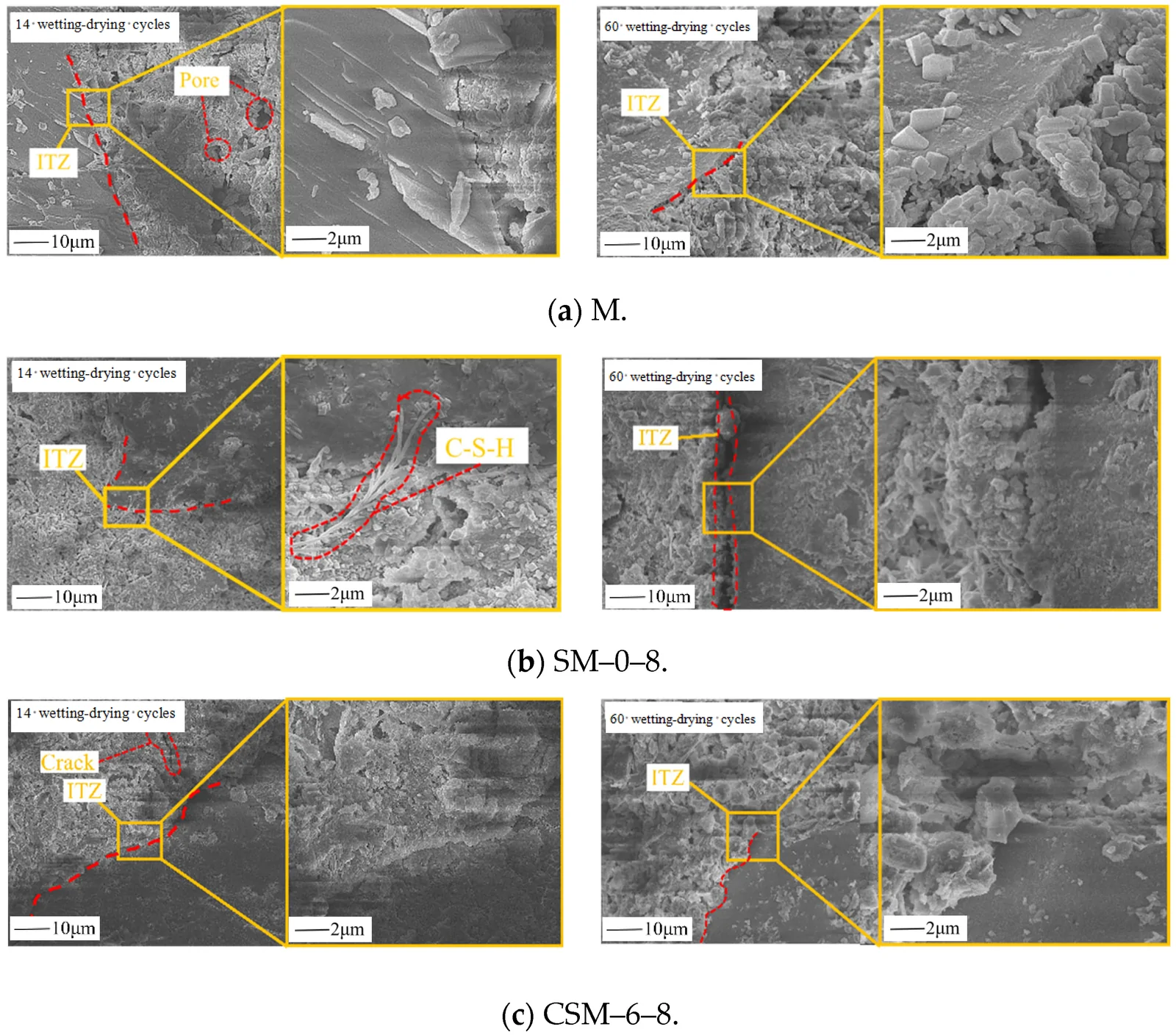
SEM images showing the ITZ morphology of modified CGC under wetting–drying cycles.

For CGC prepared using different modification methods, the ITZ structure changed markedly as the number of wetting–drying cycles increased, as shown in Figure 14. Pronounced deterioration occurred in the ITZ between the CGA and the cementitious matrix, accompanied by the formation of distinct cracks. Under wetting–drying conditions, repeated water ingress and evaporation generated expansive pressure and shrinkage stress within the capillary pores. The accumulated stresses tended to concentrate in the ITZ, thereby inducing interfacial debonding and the initiation and propagation of microcracks. This deterioration was manifested by obvious separation between the CGA and the cement paste [46].

As the wetting–drying cycles progressed, varying degrees of cracking were observed in the ITZ of all specimens. After 60 wetting–drying cycles, the ITZ of the unmodified M specimen exhibited pronounced cracking. This may be associated with the loose and porous surface, relatively low hardness, and high water absorption of the CGA, as indicated by the corresponding physical-property results. These characteristics weakened the interfacial bonding between the aggregate and the cementitious matrix. Consequently, the ITZ was more susceptible to cracking under wetting–drying cycles. As shown in Figure 14b, after 14 wetting–drying cycles, the ITZ of SM–0–8 exhibited a relatively compact morphology with fewer visible interfacial defects. This improvement may be associated with sodium silicate modification, which may have contributed to an alkaline environment and promoted subsequent reaction processes. These conditions may have promoted secondary reaction processes and the formation of additional gel-like hydration products within the ITZ, which may have contributed to pore filling and improved ITZ compactness. As shown in Figure 14c, after 60 wetting–drying cycles, CSM–6–8 exhibited a relatively dense ITZ microstructure, with few obvious microcracks or pores observed at the aggregate–paste interface. This phenomenon may be associated with the formation of an epoxy-related surface coating after curing, which may have contributed to the observed improvement in ITZ morphology. On the one hand, the epoxy-associated coating may have partially covered or sealed residual cracks and pores remaining after sodium silicate modification. On the other hand, the observed coating morphology is consistent with a possible sealing or bridging contribution within the ITZ, which may have contributed to improved ITZ compactness. Consequently, the ITZ exhibited a relatively denser microstructural morphology.

**Figure 15:**
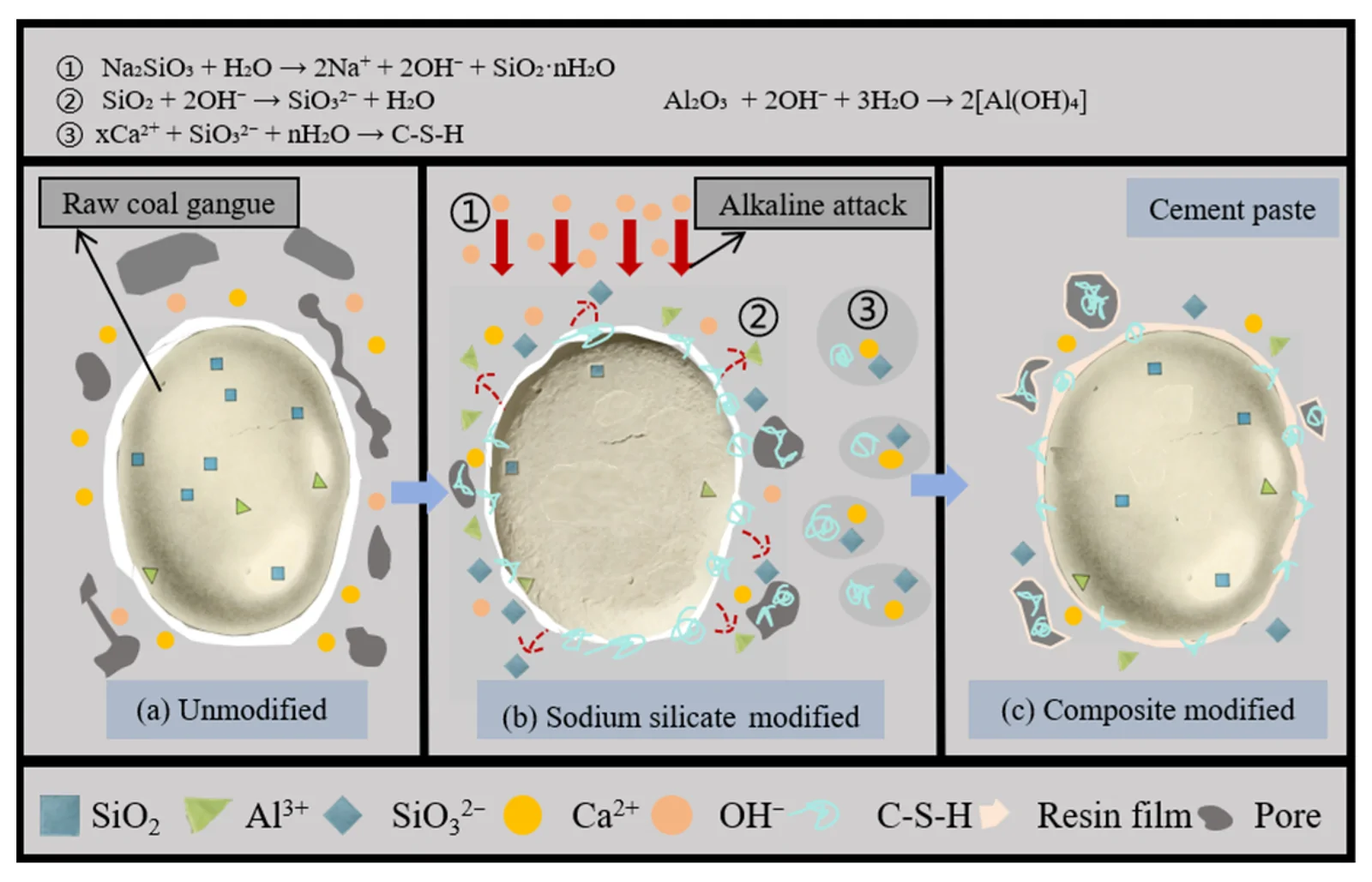
Proposed ITZ densification mechanism under combined chemical–physical modification.

To further elucidate the modification mechanism of CGC, schematic diagrams were constructed to illustrate the ITZ modification mechanisms of CGC subjected to single and combined modification. As shown in Figure 15a, the unmodified concrete specimen exhibited numerous pores on the aggregate surface and a relatively loose and defective ITZ. As shown in Figure 15b, in the specimen modified with sodium silicate alone, sodium silicate hydrolysis released abundant OH^−^, thereby creating an alkaline environment. This alkaline condition may promote the dissolution of potentially reactive Si- and Al-bearing phases on the coal gangue surface, thereby releasing soluble silicate and aluminate species. Meanwhile, Ca-containing species in the cement paste may interact with dissolved silicate species near the CGA surface, potentially contributing to the formation of additional C-S-H-type gel within the ITZ. The possible formation of additional gel-like hydration products may contribute to partial pore filling and reduced visible interfacial defects, thereby improving ITZ compactness. As shown schematically in Figure 15c, for the concrete subjected to combined chemical–physical modification, the epoxy-associated coating is proposed to provide an additional sealing effect on residual pores and voids remaining after sodium silicate modification. The combined contributions of these two proposed modification mechanisms may have contributed to further densification of the ITZ structure. These schematic diagrams summarize the proposed mechanisms that may contribute to ITZ improvement in CGC subjected to single and combined modification.

### 3.6. Assessment of Low-Carbon Resource Utilization Benefits

Coal gangue, a typical industrial solid waste generated during coal mining and washing, occupies large areas of land when stockpiled for long periods and may also cause dust emissions, leachate pollution, and potential ecological risks. After crushing and screening, coal gangue can be used as a coarse aggregate in concrete to partially replace natural crushed stone, thereby reducing the energy consumption and carbon emissions associated with the extraction, crushing, screening, and transportation of natural aggregates. Therefore, CGC not only provides an effective pathway for the resource utilization of solid waste but also shows considerable potential for the development of low-carbon construction materials. According to the mix design, the replacement ratio of coal gangue coarse aggregate was 25%. Compared with conventional concrete, the amount of natural crushed stone used per cubic meter of CGC decreased from 1038 kg to 778.5 kg, corresponding to a reduction of 259.5 kg of natural crushed stone per cubic meter. To further evaluate the carbon-reduction potential of replacing natural coarse aggregate with coal gangue coarse aggregate at a replacement ratio of 25%, the carbon emission reduction was calculated according to Equation (6), and the corresponding result is presented in Equation (11). From the perspective of coarse aggregate substitution, the production of 1 m^3^ of CGC can reduce carbon emissions by approximately 0.52 kg CO_2_-eq. Although the direct carbon-reduction benefit of replacing natural coarse aggregate with coal gangue coarse aggregate is relatively limited, this approach offers comprehensive low-carbon advantages in terms of solid waste resource utilization and natural aggregate conservation.

It should be emphasized that the estimated reduction of approximately 0.52 kg CO_2_-eq/m^3^ represents only the carbon-reduction benefit associated with replacing natural coarse aggregate with coal gangue coarse aggregate and does not represent the net carbon footprint reduction of the complete combined-modification process. The production of sodium silicate, waterborne epoxy resin, and curing agent introduces additional embodied carbon, while the associated treatment processes also involve additional material and energy inputs. Therefore, a complete evaluation of the environmental performance should consider both the carbon-reduction benefit of natural aggregate substitution and the additional burdens associated with the modification process. At the same time, the improved mechanical performance and resistance to wetting–drying deterioration achieved by the combined modification may contribute to a longer service life and reduced maintenance or replacement requirements, providing potential lifecycle environmental benefits. Future studies should conduct a comprehensive lifecycle assessment incorporating modifier production, actual modifier consumption, processing energy, and potential service-life benefits to determine the overall environmental performance of the combined-modification strategy.

(11) $$\Delta C_{1}=m_{NA}\cdot EF_{NA}-m_{CGA}\cdot EF_{CGA}\;\;\Delta C=259.5\times (0.004-0.002)=0.519\;\mathrm{kg}\;{\mathrm{CO}}_{2}{\text{-}\mathrm{eq}/m}^{3}$$

## 4. Discussion

### 4.1. Effect of Single Modification on the Wetting–Drying Resistance of CGC

Under wetting–drying cycles, CGC is prone to progressive deterioration, and its overall performance is generally inferior to that of conventional concrete. This is mainly attributed to the connected pores and microcracks within CGA, which may be inherent or generated during crushing and screening. These defects increase the water absorption of CGA and reduce their structural compactness, thereby weakening the resistance of CGC to deterioration under wetting–drying cycles.

**Figure 16:**
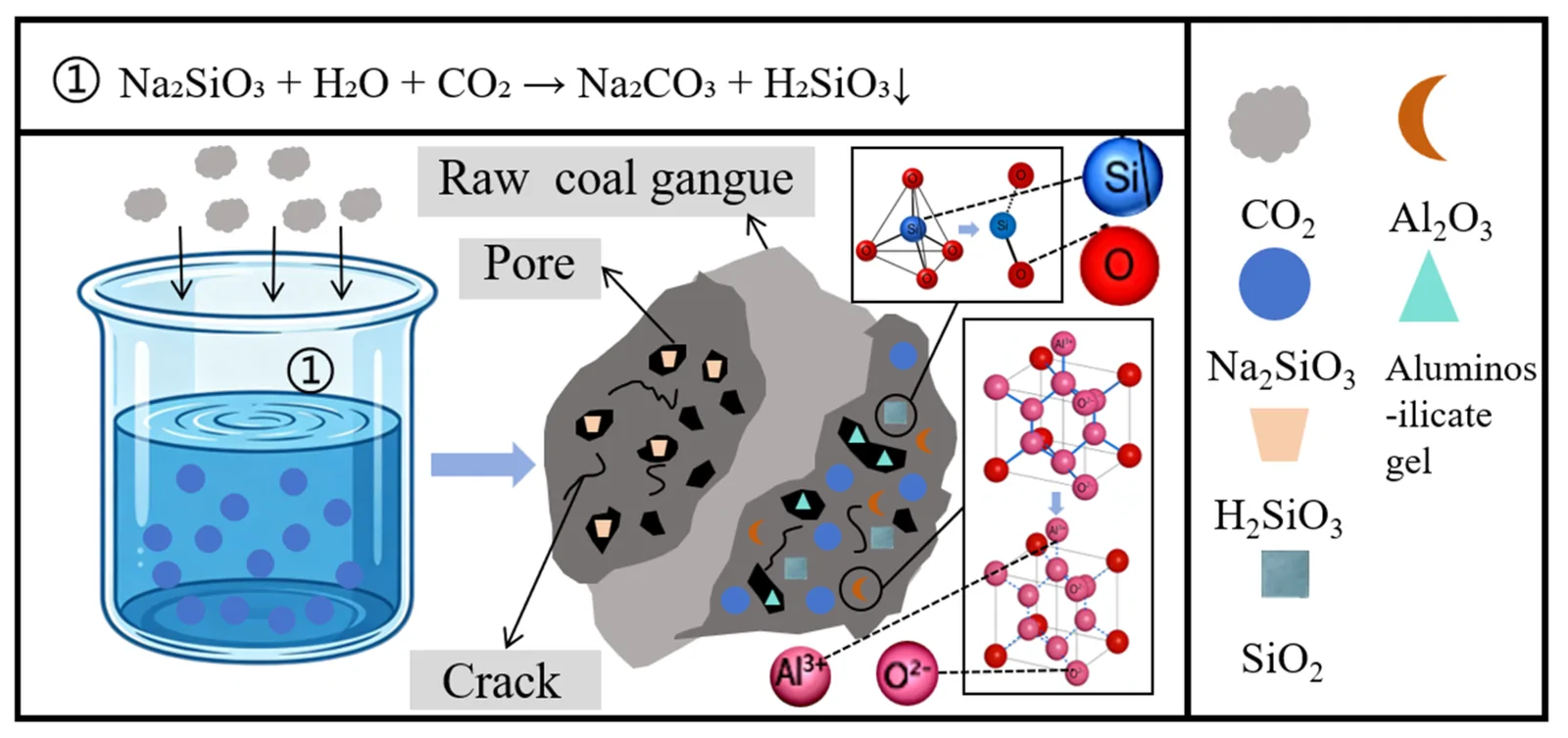
Proposed multi-scale enhancement mechanism induced by sodium silicate modification.

After modification with sodium silicate solution, SM–0–8 exhibited a higher relative dynamic modulus of elasticity, compressive strength, and flexural strength than M. This improvement may be associated with three mutually reinforcing mechanisms proposed for sodium silicate modification. First, sodium silicate modification may promote hydration-related and secondary reaction processes, potentially contributing to the formation of additional gel-like hydration products that may partially fill capillary pores and microcracks and improve matrix compactness. Second, sodium silicate solution exhibits good permeability and can penetrate the interior of CGA through capillary action [47]. The OH^−^ released from sodium silicate may promote the cleavage of Si-O-Si and Si-O-Al bonds in susceptible Si- and Al-containing phases in coal gangue, thereby facilitating the release of soluble silicate and aluminate species. These dissolved Si- and Al-containing species may subsequently participate in condensation and secondary reaction processes, potentially contributing to the formation of aluminosilicate gel-like products and to the early-stage performance development observed during wetting–drying exposure, as schematically illustrated in Figure 16. In addition, sodium silicate may react with CO_2_ from the air to form H_2_SiO_3_^−^ related precipitates, which may contribute to surface deposition on the aggregate, as schematically illustrated by reaction ① in Figure 16. This process seals surface pores and reduces water absorption, thereby further enhancing the internal compactness of the specimens. Overall, these three mechanisms jointly contribute to SM–0–8’s improved resistance to deterioration under wetting–drying cycles. Figure 16 presents a comprehensive schematic illustration of the modification mechanism of CGC, highlighting the key role of sodium silicate in improving the microstructure and performance of the specimens. Nevertheless, although sodium silicate modification alone markedly improved specimen performance, small residual pores and internal defects remained in the matrix, indicating that the improvement achieved by sodium silicate alone was limited.

### 4.2. Enhancement Mechanism of Combined Chemical–Physical Modification

As the number of wetting–drying cycles increased, the groups subjected to combined modification exhibited more pronounced advantages over the group modified with sodium silicate alone. After 120 wetting–drying cycles, the compressive and flexural strengths of CSM–6–8 were 11.92% and 33.70% higher than those of SM–0–8, respectively, as shown in Figure 8 and Figure 9. When the dosage of waterborne epoxy resin was 3 wt.%, the aggregate surface exhibited a relatively discontinuous coating morphology, with locally exposed areas remaining, as shown in Figure 5c, which may have limited the overall modification effect. In contrast, when the dosage of waterborne epoxy resin was increased to 9 wt.%, local agglomeration-like features were observed, as shown in Figure 5e, which may be associated with the higher resin dosage and the curing process. This resulted in uneven resin film thickness, which weakened the bond between the cement paste and aggregate, thereby causing a downward trend in mechanical properties during the later stage of wetting–drying cycles.

Notably, the flexural strength was more sensitive to the resin dosage than the compressive strength, as shown in Figure 8 and Figure 9. After 120 wetting–drying cycles, CSM–6–8 containing 6 wt.% waterborne epoxy resin showed increases of 11.92% and 33.70% in compressive and flexural strengths, respectively, compared with SM–0–8. The percentage increase in flexural strength was approximately 2.83 times that of compressive strength. This difference can be attributed to the more direct contribution of the bridging effect of the resin film to the flexural resistance of the specimens. Overall, compared with SM–0–8, CSM–3–8, CSM–6–8, and CSM–9–8 exhibited more pronounced improvements in compressive strength, flexural strength, relative dynamic modulus of elasticity, and ITZ compactness. These findings indicate that, within the investigated dosage range, the performance of the combined modification appeared to depend on the balance between coating development and the tendency toward resin agglomeration rather than simply on increasing the polymer dosage. The 6 wt.% resin dosage exhibited the best overall performance among the investigated dosage levels under the present mix design and the 25% coal gangue coarse aggregate replacement ratio. More importantly, the results reveal a clear dosage-dependent effect of waterborne epoxy resin on the combined modification performance. Although the exact best-performing dosage may vary with mix proportions and aggregate replacement ratios, the observed trend provides useful guidance for optimizing similar combined-modification systems. These results indicate that CGC subjected to combined modification exhibited superior overall performance compared with CGC modified with sodium silicate alone. After 120 wetting–drying cycles, CSM–6–8 still retained a performance advantage over SM–0–8, suggesting that the epoxy-associated coating may remain effective during the investigated exposure period. Under prolonged exposure, repeated wetting–drying and interfacial stresses may gradually affect the integrity and adhesion of the coating. Therefore, longer-term exposure tests and direct characterization of the coating are needed to further verify its stability.

These findings should be interpreted within the scope of the present experimental conditions. The performance evolution during the first 28 wetting–drying cycles may be influenced by both material maturation and modification-related microstructural development. Differences also exist between the controlled laboratory wetting–drying regime and actual field environments, where moisture variations are often accompanied by temperature fluctuations. In addition, the proposed modification mechanism is supported by the combined XRD, SEM, and EDS observations, which provide evidence for possible gel-like hydration products and an epoxy-associated surface coating. Future studies may further incorporate age-matched non-cyclic reference specimens, investigate the combined effects of wetting–drying cycles and temperature fluctuations, and employ complementary characterization techniques to further verify the proposed mechanism and better represent actual service conditions.

From the perspective of practical implementation, the combined modification process is relatively simple and does not require highly specialized equipment. However, the additional modifier cost, two-stage immersion, and 7 d air-drying period may increase processing time and cost at an industrial scale. Further optimization of modifier dosage, treatment duration, and process continuity, together with pilot-scale and techno-economic evaluation, is therefore needed prior to large-scale application.

## 5. Conclusions

In this study, wetting–drying cycle tests were conducted to systematically investigate the effects of single sodium silicate modification and combined chemical–physical modification on the wetting–drying resistance of CGC. A multi-scale analytical approach integrating SEM-based microstructural characterization and XRD phase analysis was employed to investigate the possible underlying modification mechanisms. The main findings are summarized as follows:

1. After 60 wetting–drying cycles, CSM–6–8 exhibited compressive and flexural strengths of 50.5 MPa and 10.9 MPa, respectively. After 120 cycles, its compressive and flexural strengths remained 11.92% and 33.70% higher than those of SM–0–8, respectively, indicating that the combined chemical–physical modification provided a sustained improvement in the wetting–drying resistance of CGC. After 90 wetting–drying cycles, the relative dynamic modulus of elasticity of CSM–6–8 decreased by only 3.4% relative to its peak value at 28 cycles.
2. Under wetting–drying cycles, the microstructural observations were consistent with possible pore filling associated with sodium silicate-related gel-like reaction products and additional sealing associated with the cured waterborne epoxy resin, which together may have contributed to improved matrix compactness and resistance to deterioration under wetting–drying cycles.
3. Replacing 25% of natural coarse aggregate with coal gangue coarse aggregate reduced natural crushed stone consumption by 259.5 kg/m^3^ and carbon emissions by approximately 0.52 kg CO_2_-eq/m^3^, indicating its potential value in solid waste recycling and green building material development.

More importantly, this study proposes a combined chemical–physical modification strategy for porous coal gangue aggregates, providing a mechanistic framework for designing durable recycled aggregate concrete exposed to cyclic environmental actions.

## Figures and Tables

**Table 1:** Chemical compositions of cement and coal gangue (mass fraction, %).

Chemical Composition	Al_2_O_3_	SiO_2_	Fe_2_O_3_	CaO	MgO	K_2_O	Na_2_O	Other Components
Coal gangue	29.1	55.75	6.7	3.9	1.4	0.8	1.3	1.05
Cement	8.49	24.19	2.85	59.33	3.92	0.44	0.23	0.55

**Table 2:** Physical properties of waterborne epoxy resin.

Type	Density	pH Value	Epoxy Value	Particle Size (μm)	Minimum Film-Forming Temperature (°C)	Solid Content (%)
DY-128-50	1.05	6–8	0.52	<0.3	7	50 ± 3

**Table 3:** Physical properties of curing agent.

Type	Density (g/cm^3^)	pH Value	Appearance	Active Ingredient Content (wt.%)	Active Hydrogen Equivalent	Viscosity (mPa·s, 25 °C)
DY-175	1.05–1.08	9.5–10.5	<4	49.0–51.0	330	6500–9000

**Table 4:** Modification schemes for coal gangue coarse aggregates.

Specimen ID	Coal Gangue Replacement Ratio	Modification Type	Modification Scheme
N	0	Unmodified	Natural aggregate
M	25%	Unmodified	Raw coal gangue
SM–0–8	25%	Single modification	8 wt.% sodium silicate modification
CSM–3–8	25%	Combined chemical–physical modification	8 wt.% sodium silicate + 3 wt.% waterborne epoxy resin modification
CSM–6–8	25%	Combined chemical–physical modification	8 wt.% sodium silicate + 6 wt.% waterborne epoxy resin modification
CSM–9–8	25%	Combined chemical–physical modification	8 wt.% sodium silicate + 9 wt.% waterborne epoxy resin modification

**Table 5:** Mix proportions of CGC (kg/m^3^).

Group	Water	Cement	CGA	Crushed Stone	Fly Ash	Fine Aggregate	Water-Reducing Admixture
Ordinary concrete	190	430	0	1038	47	770	5
CGC	190	430	259.5	778.5	47	770	5

Note: The mix proportions of CGC apply to all SM and CSMs. The mixture parameters are identical among these groups, with the only difference being the modification treatment applied to the CGA.

## Data Availability

The raw data supporting the conclusions of this article will be made available by the authors on request.
